# *Helicobacter pylori *SlyD stabilizes TPT1 via hnRNPK and enhances OCT1-mediated CDX2 transcriptional activation to drive gastric intestinal metaplasia

**DOI:** 10.1186/s12916-025-03911-8

**Published:** 2025-02-06

**Authors:** Shuwen Zheng, Yingying Wang, Chuxuan Ni, Rui Guo, Xunan Qiu, Jijun Chen, Lu Wang, Xiaohu Sun, Moye Chen, Yunen Liu, Yuan Yuan, Yuehua Gong

**Affiliations:** 1 Tumor Etiology and Screening Department of Cancer Institute and General Surgery, the First Hospital of China Medical University, Shenyang, 110001 China; 2 Key Laboratory of Cancer Etiology and Prevention in Liaoning Education Department, the First Hospital of China Medical University, Shenyang, 110001 China; 3 Key Laboratory of GI Cancer Etiology and Prevention in Liaoning Province, the First Hospital of China Medical University, Shenyang, 110001 China

**Keywords:** *H. pylori*, SlyD, Gastric intestinal metaplasia, CDX2, Gastric precancerous disease, Dihydroartemisinin

## Abstract

**Background:**

Gastric intestinal metaplasia (GIM) represents an important precancerous lesion in intestinal-type gastric cancer, triggered by persistent *Helicobacter pylori* (*H. pylori*) infection. In a previous study, we unveiled SlyD as a novel virulence factor of *H. pylori*, establishing its role in GIM induction through TPT1. However, the underlying mechanism remains undetermined.

**Methods:**

Gastric epithelial cells were stimulated with *H. pylori* 26695, a SlyD inactivated mutant (ΔSlyD), and purified HpSlyD protein, respectively. Real-time qPCR and western blot were subsequently used to assess the expression levels of hnRNPK, TPT1, OCT1, and GIM markers. RNA sequencing was employed to identify differentially expressed genes associated with *H. pylori* SlyD infection. Protein stability was evaluated using cycloheximide. Molecular interactions were investigated through co-immunoprecipitation, chromatin immunoprecipitation, and dual-luciferase reporter assays. Additionally, molecular docking was utilized to predict TPT1 inhibitors. Immunohistochemistry staining was conducted to validate hnRNPK, TPT1, OCT1, and CDX2 expression in gastric tissue samples from both human and Mongolian gerbils.

**Results:**

*H. pylori* SlyD upregulates TPT1 and induces the expression of GIM markers through hnRNPK. The interaction between hnRNPK and TPT1 enhances TPT1 protein stability, with *H. pylori* SlyD intensifying this association. TPT1 promotes the expression of GIM markers mediated via OCT1, which binds to CDX2 promoter region, thereby modulating its transcriptional activity. Dihydroartemisinin has the potential to target TPT1, inhibiting the *H. pylori* SlyD-induced expression of GIM markers.

**Conclusions:**

In vitro and in vivo experiments verified that *H. pylori* SlyD enhances TPT1 stability through hnRNPK, leading to OCT1-mediated transcriptional activation of CDX2 and the initiation of the GIM process. Our study offers novel perspectives on the pathogenesis of *H. pylori*-related gastric precancerous conditions.

**Supplementary Information:**

The online version contains supplementary material available at 10.1186/s12916-025-03911-8.

## Background

Gastric intestinal metaplasia (GIM) is a significant precancerous lesion in intestinal-type gastric cancer (IGC) [1], increasing the risk of gastric cancer (GC) by 3.6-fold [2]. The development of GIM is influenced by various risk factors, such as gender [3, 4], smoking [5], and vitamin D [6]. However, the most crucial risk factor is *Helicobacter pylori* (*H. pylori*) infection [7, 8], which colonizes the gastric mucosa, manipulating host immune responses to induce injury and inflammation, that promote GIM [9]. *H. pylori* primarily exerts its effects through virulence factors like *cagA* and *vacA*, which are closely related to *H. pylori* pathogenicity [10, 11].

In a previous study, we unveiled a novel *H. pylori* virulence factor, SlyD [12, 13]. Subsequent investigations suggested that SlyD putatively upregulated the expression of caudal type homeobox protein 2 (CDX2), by modulating translationally controlled tumor protein 1 (TPT1/TCTP) [14]. CDX2 is recognized as a pivotal driver in GIM [15] and is implicated in the regulation of other GIM markers like Villin and MUC2 [16, 17]. However, the mechanism by which *H. pylori* SlyD regulates CDX2 expression through TPT1 remains unclear.

Heterogeneous nuclear ribonucleoprotein K (hnRNPK) exhibits high expression levels in GC tissue and plays a role in driving disease progression by regulating cell proliferation, invasion, and migration [18, 19]. Additionally, hnRNPK is implicated in various disease processes through its involvement in cell differentiation [20–22]. *H. pylori* infection and hnRNPK may have synergistic effects in gastric carcinogenesis [23]. Functioning as an RNA-binding protein, hnRNPK participates in diverse biological processes such as gene transcription and translation. Moreover, hnRNPK influences downstream protein stability, contributing to the development malignant tumor [24, 25]. For instance, hnRNPK interacts with β-catenin and increases the protein stability of β-catenin in neuroblastoma [26]. While in breast cancer, hnRNPK affects ILF2 protein degradation [27]. However, the impact of hnRNPK in *H. pylori* SlyD infection within the context of GIM remains unexplored.

POU Domain, Class 2, Transcription Factor 1 (POU2F1/OCT1), is a versatile member of the POU family that plays essential roles in regulating cell differentiation and gene expression. It can function independently as a transcription factor or interact with other proteins to form regulatory complexes [28–30]. Notably, OCT1 exhibits high expression levels in various malignant tumors [31–33] and in GIM tissues [34, 35]. Nonetheless, the involvement of OCT1 in GIM processes induced by *H. pylori* SlyD remains to be elucidated.

In this study, we observed that *H. pylori* SlyD regulated hnRNPK and influenced TPT1 protein stability. Moreover, the upregulation of TPT1 led to increased OCT1 expression, subsequently activating CDX2 transcriptionally and triggering GIM. The correlation among hnRNPK/TPT1/OCT1, GIM, and *H. pylori* SlyD infection was further validated in human and Mongolian gerbils (MGs) gastric tissues. Additionally, our observations indicated that dihydroartemisinin (DHA) inhibited the expression of GIM markers induced by *H. pylori* SlyD. This finding suggests a potential reversal of GIM progression caused by *H. pylori* infection, highlights a possible strategy for preventing and treating early GC.

## Methods

### *H. pylori* strains and culture

All *H. pylori* strains were cultured under microaerobic conditions at 37 °C on brain–heart infusion plates supplemented with 10% defibrillated sheep blood. The *H. pylori* 26695 was acquired from the American Type Culture Collection (ATCC, MD, USA). A SlyD inactivated mutant strain, where chloramphenicol-resistant fragments replaced *slyD* fragments (referred to as ΔSlyD in this article), was generated based on *H. pylori* 26695 [12]. *H. pylori* strain 1 (*HpslyD* negative) and *H. pylori* strain 2 (*HpslyD* positive) were isolated from human gastric tissues [36]. *H. pylori* was identified through characteristic colony morphology, Gram staining, and urease tests.

### Cell culture and stimulation

Gastric epithelial cells AGS and GES-1 were purchased from the Chinese Center for Conservation of Typical Cultures (CCTCC, Wuhan, China). The cells were treated with *H. pylori* 26695 and ΔSlyD for 24 h at a multiplicity of infection (MOI) = 100, respectively. Purified HpSlyD protein (200 ng/mL, Sino Biological, Beijing, China), with or without 10 μM DHA (MedChemExpress, NJ, USA), was utilized to stimulate the cells for 40 h.

### Small interfering RNA (siRNA), plasmid, and lentivirus transfections

Specific siRNA targeting hnRNPK was provided by Sangon Biotech Co. (Shanghai, China), with sequences detailed in Additional file 1: Table S1. The OCT1 expression plasmid (GV740-POU2F1), TPT1 overexpression lentivirus (LV-TPT1), and TPT1 shRNA lentivirus (LV-TPT1-RNAi) were obtained from Genechem Co. (Shanghai, China). Transfection of all siRNAs and plasmids into cells was carried out using jetPRIME transfection reagent (Polyplus, France), while lentiviruses were transfected using Polyberene transfection reagent (Millipore, MA, USA). Stably transfected cell lines were selected using 1 μg/ml puromycin for 7 days.

### RNA extraction and real-time quantitative PCR (RT-qPCR)

RNA extraction was carried out using TRNzol Universal Reagent (TIANGEN, Beijing, China). Subsequently, RT-qPCR was performed using SYBR Premix Ex Taq II (Takara, Dalian, China). Relative mRNA gene expression levels were calculated utilizing the 2^−ΔΔCt^ method. Primer sequences for RT-qPCR can be found in Additional file 1: Table S1.

### Western blot

Total proteins were extracted using RIPA buffer (Solarbio, Beijing, China) containing 1% phenylmethanesulfonyl-fluoride protease inhibitor. Western blot analysis was conducted following standard procedures. Visualization of proteins was accomplished with an Ultra-Sensitive Enhanced Chemiluminescence Kit (Beyotime, Shanghai, China) and ImageJ (National Institutes of Health, MD, USA) was employed for quantifying protein levels. Antibodies used in western blot analysis are listed in Additional file 1: Table S2.

### RNA sequencing (RNA-seq)

AGS cells were exposed to *H. pylori* 26695 or △SlyD for 24 h (MOI = 100). Subsequently, total RNA was extracted according to above method, and mRNA sequencing was performed on the Illumina Hi-seq platform (Illumina, CA, USA). RNA libraries were prepared using KC-DigitalTM Stranded mRNA Library Prep Kit for Illumina® (Wuhan Seqhealth, Wuhan, China) following the manufacturer’s protocols. FASTP (version 0.23.0) software was used for data quality control, including removing adapter sequences from the reads, filtering out reads shorter than 15 bp or low-quality reads (the proportion of bases with a quality score below Q20 > 0.08), and discarding reads containing excessive undefined bases. The RPKM value was used as a metric for assessing gene expression levels. The sequencing data used in this study has been deposited in NCBI’s Gene Expression Omnibus (GEO) database (http://www.ncbi.nlm.nih.gov/geo/) and is available under accession number GSE282336.

### Co-immunoprecipitation (Co-IP)

For Co-IP experiments, an immuno(co-)precipitation kit (Absin, Shanghai, China) was employed. Cell lysates were incubated overnight with TPT1 and IgG antibodies along with protein A/G beads on a rocking platform at 4 °C. Following this incubation, the beads were precipitated by centrifugation, washed three times in IP lysis buffer, and purified protein complexes were obtained. Western blot analysis was performed after resuspending beads in loading buffer. Antibodies used in Co-IP analysis are listed in Additional file 1: Table S2.

### Protein stability assay

Cells were exposed to cycloheximide (CHX) at a concentration of 80 μg/mL. Total protein was extracted from the cells every 3 h, with 10 μg/mL puromycin added to the cells 20 min prior to extraction. The expression of the target protein was analyzed using western blot, with puromycin antibody employed to monitor the synthesis of nascent proteins. GAPDH was used as a normalizing control during the experiment.

### Bioinformatics analysis

Functional and signaling pathway enrichment analyses were conducted using R-4.3.3 software, leveraging the Gene Ontology (GO, https://www.geneontology.org/) [37] and Kyoto Encyclopedia of Genes and Genomes (KEGG, https://www.kegg.jp/) databases [38]. The UCSC genome browser (https://genome.ucsc.edu/) was utilized to predict transcription factors potentially binding to gene promoters [39]. The GEPIA2.0 database (http://gepia2.cancer-pku.cn/) was employed to investigate correlations between gene expression in diseases [40]. Furthermore, the JASPAR database (https://jaspar.genereg.net/) was used to predict molecular binding sites between molecules [41].

### Dual-luciferase reporter assay

The region spanning from − 2000 to 0 nt of the CDX2 promoters (including wild-type and mutants) were amplified and cloned into pGL3-basic vectors. HEK293 cells were then transfected with the pGL3-CDX2 constructs, OCT1 expression plasmid (GV740-POU2F1), or its control plasmid, along with an internal reference vector pRL-TK, using X-tremegene HP transfection reagent (ROCHE, Basel, Switzerland) for 24 h. Subsequently, the relative luciferase activity was assessed in the cells using the Dual-Luciferase® Reporter Assay System (Promega, Beijing, China).

### Chromatin immunoprecipitation (ChIP) assay

A ChIP assay was performed using a SimpleChIP® Plus Enzymatic Chromatin IP kit (CST, MA, USA) according to the manufacturer’s instructions. Following immunoprecipitation and DNA extraction, the DNA samples were analyzed using RT-qPCR. Antibodies used in ChIP analysis are listed in Additional file 1: Table S2.

### Molecular docking

To identify potential drug candidates targeting the TPT1 protein, the secondary structures of small molecules were retrieved from the PubChem database (https://pubchem.ncbi.nlm.nih.gov/) [42], while three-dimensional (3D) protein structures were obtained from the Protein Data Bank (PDB, https://www.wwpdb.org/) [43]. The structures of both small molecules and proteins were preprocessed using Chem3D 19.0. Protein structures were further prepared for molecular docking using AutoDockTools 1.5.6, including the removal of water molecules and the addition of hydrogen atoms. Molecular docking simulations were then conducted. The docking results were analyzed and visualized using the Protein–Ligand Interaction Profiler (PLIP) web tool (https://plip-tool.biotec.tu-dresden.de/plip-web/) [44] and PyMOL 2.5.2 software. PLIP was utilized to identify and characterize binding sites, while PyMOL was employed to annotate binding site residues.

### Human tissue specimens and *H. pylori* SlyD detection

A total of 70 paraffin-embedded tissue specimens from human gastric mucosa were collected, including 18 patients with chronic non-atrophic gastritis (GS) and 52 patients with GIM. The study was approved by the Ethics Committee of the First Hospital of China Medical University (No.AF-SOP-07–1.0–01), and informed consent was obtained from all patients.

DNA extraction from the tissues was carried out using the WaxFreeTM DNA kit (Quick DNA preparation for FFEP; TrimGen Corp., MD, USA). PCR was employed to detect the presence of *16S rRNA*, *glmM*, and *slyD* genes. *H. pylori* infection status was determined based on the positivity of *H. pylori glmM* and *16S rRNA* genes. A patient was classified as *H. pylori* positive if both genes were positive. Amplification of the conserved region of the *slyD* gene was performed to ascertain if a patient was *HpslyD* positive. Primer sequences of the PCR are detailed in Additional file 1: Table S1.

### *H. pylori* SlyD infection of MGs

MGs were infected with *H. pylori* strains following the protocol outlined by Liu et al. [36]. Specific pathogen-free MGs (6 weeks old males) were fasted from food and water for 16 h before oral gavage. A total of 15 MGs were divided into three groups: one group received *H. pylori* strain 1 at a concentration of 10^8^ colony-forming units (CFU) in 1 mL, another group was treated with *H. pylori* strain 2 at the same concentration, and the third group received 1 mL of saline as the control group via gavage administration. After a duration of 73 weeks, all animals underwent complete autopsy procedures, and gastric tissues were collected, fixed, and embedded in paraffin for further analyses.

### Immunohistochemistry (IHC) Staining

Following formaldehyde fixation, paraffin-embedded human and MG gastric tissues were processed for IHC staining according to the manufacturer’s protocols (Maxim, Fuzhou, China). Primary antibodies used in the IHC procedure are listed in Additional file 1: Table S2. Photomicrographs were captured using an upright microscope (Nikon Eclipse Ni-U, Tokyo, Japan). Subsequently, two pathologists, who were blinded to the clinical information, independently assessed and scored the samples according based on the scoring method described by Li et al. [14].

### Statistical analysis

Statistical analyses were performed using SPSS 25.0 and GraphPad Prism 9.0.0. Quantitative data from a minimum of three independent experiments were presented as the mean ± SD. Student’s *t* tests were employed to evaluate differences between groups, while chi-square tests were utilized for comparing categorical data. Spearman’s correlation tests were performed to analyze correlations between various continuous variables. A significance level of *P* < 0.05 was set to determine statistical significance in all analyses.

## Results

### *H. pylori* SlyD induces the expression of GIM markers by upregulating TPT1 protein levels

**Fig. 1:**
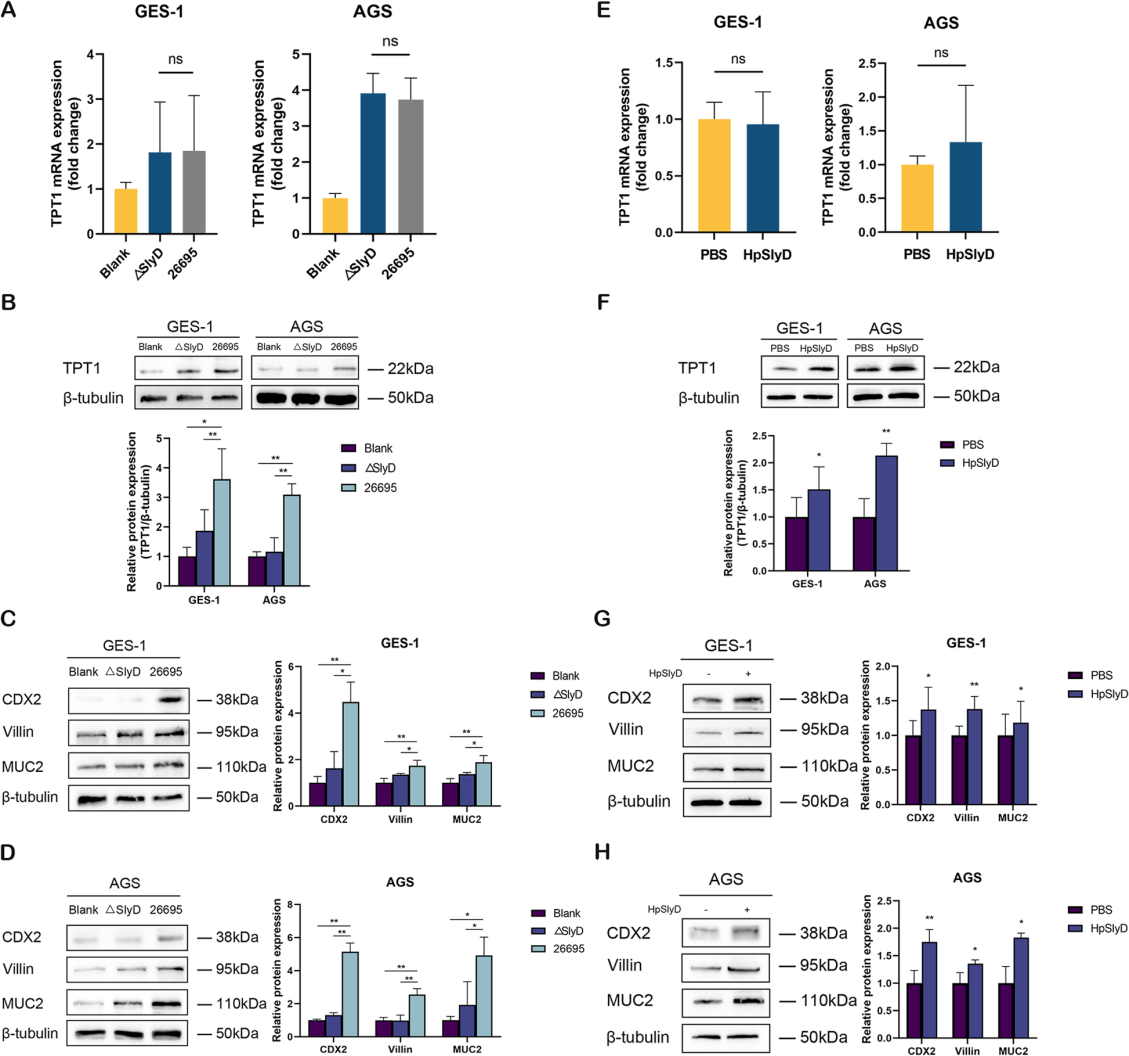
*H. pylori* SlyD induces GIM marker expression by upregulating TPT1 protein levels. **A** mRNA expression of TPT1 in *H. pylori* SlyD-stimulated GES-1 and AGS cells. GAPDH was used as an internal control. **B **Western blot for TPT1 expression in *H. pylori* SlyD-stimulated GES-1 and AGS cells. **C **Western blot for the expression of GIM markers in *H. pylori* SlyD-stimulated GES-1 cells. **D **Western blot for the expression of GIM markers in *H. pylori* SlyD-stimulated AGS cells. **E **mRNA expression of TPT1 in AGS and GES-1 cells following stimulation with purified HpSlyD protein (200 ng/mL) for 40 h. **F **Western blot for TPT1 expression in GES-1 and AGS cells after 40 h stimulation with purified HpSlyD protein (200 ng/mL). **G **Western blot for the expression of GIM markers in GES-1 cells after HpSlyD stimulation (200 ng/mL). **H **Western blot for the expression of GIM markers in AGS cells after HpSlyD stimulation (200 ng/mL). β-tubulin was used as the loading control. Significance levels: *P < .05, ** *P* < .01, *** *P* < .001, ns: non-significant

In this study, we used both *H. pylori* 26695 and ΔSlyD strains to stimulate human gastric epithelial cells in order to examine their effects on the expression of TPT1 and GIM markers, specifically CDX2, Villin, and MUC2. RT-qPCR analysis revealed no significant differences in TPT1 mRNA levels between cells infected with the *H. pylori* 26695 and those infected with the ΔSlyD strain (Fig. 1A). However, a notable increase in TPT1 protein expression was observed in cells infected with *H. pylori* 26695 (Fig. 1B). Additionally, the increase in TPT1 protein levels correlated with a concomitant rise in the expression of GIM markers (Fig. 1C and D).

Furthermore, we stimulated gastric epithelial cells with 200 ng/ml of purified HpSlyD protein. Consistent with above results, TPT1 mRNA levels remained unchanged following HpSlyD stimulation (Fig. 1E). However, we observed a significant increase in TPT1 protein levels post-stimulation with purified HpSlyD protein (Fig. 1F). In addition to the upregulation of TPT1 protein, we also detected an increase in the expression of GIM markers CDX2, Villin, and MUC2 after treatment with purified HpSlyD protein (Fig. 1G and H). Overall, our results suggest that while *H. pylori* SlyD does not directly affect TPT1 mRNA expression, it plays a significant role in enhancing TPT1 protein levels. The upregulation of TPT1 protein is associated with increased GIM marker expression, indicating that *H. pylori* SlyD influences the metaplastic transformation of gastric epithelial cells through a post-transcriptional mechanism that targets TPT1.

### *H. pylori* SlyD upregulates TPT1 protein expression via hnRNPK

To further explore the molecular mechanisms underlying *H. pylori* SlyD regulation of TPT1 protein expression, AGS cells infected with *H. pylori* 26695 and ΔSlyD strains were subjected to RNA-seq analysis. A comparison between the two strains revealed the upregulation of 3200 genes in *H. pylori* 26695 (Additional file 1: Table S3). GO analysis unveiled the involvement of *H. pylori* SlyD in biological processes such as histone modification, nuclear envelope and cell-substrate junction (Additional file 2: Fig S1). Furthermore, KEGG enrichment analysis highlighted the participation of *H. pylori* SlyD in pathways like human papillomavirus infection, endocytosis, regulation of actin cytoskeleton and other signaling pathways (Additional file 2: Fig S2).

**Fig. 2:**
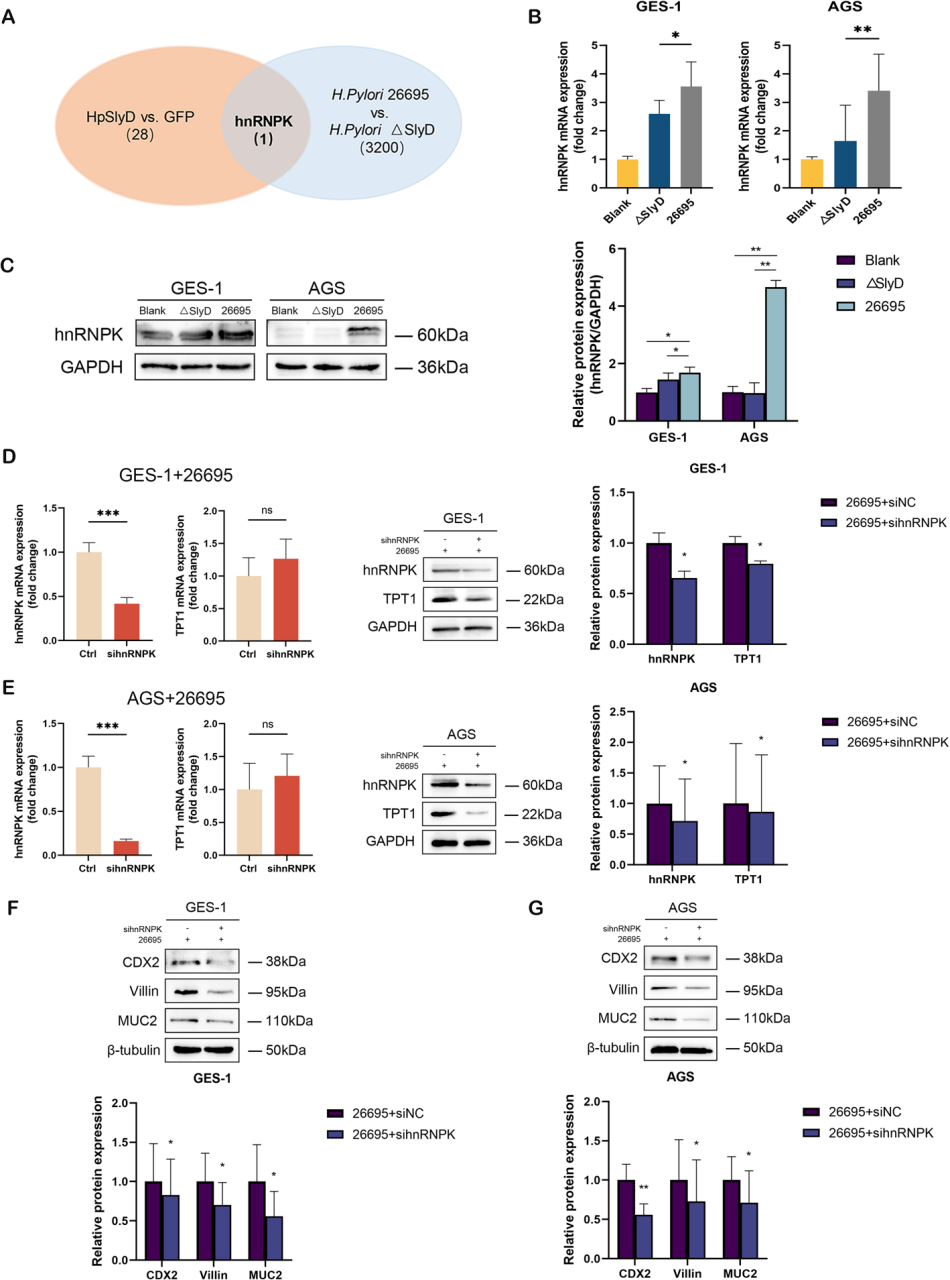
*H. pylori* SlyD upregulates TPT1 protein expression through hnRNPK and promotes GIM marker expression. **A** RNA-seq data from co-cultured AGS cells with *H. pylori* 26695 and △SlyD strain for 24 hours were integrated with two-dimensional gel electrophoresis mass spectrometry data from HpSlyD-GFP stable cell line to identify intersection genes hnRNPK. **B **The mRNA expression of hnRNPK was analyzed in *H. pylori* SlyD-stimulated GES-1 and AGS cells. GAPDH was used as internal control. **C** Western blot for the expression of hnRNPK in *H. pylori* SlyD-stimulated GES-1 and AGS cells. Protein band intensity was normalized to GAPDH band intensity. **D** RT-qPCR and western blot of hnRNPK and TPT1 expression, respectively, in *H. pylori* SlyD-stimulated GES-1 cells treated with sihnRNPK. Protein band intensity was normalized to GAPDH band intensity. **E **RT-qPCR and western blot of hnRNPK and TPT1 expression, respectively, in *H. pylori* SlyD-stimulated AGS cells with sihnRNPK treatment. Protein band intensity was normalized to GAPDH band intensity. **F** Western blot for the expression of GIM markers in *H. pylori* SlyD-stimulated GES-1 cells with sihnRNPK treatment. **G **The expression of GIM markers in *H. pylori* SlyD-stimulated AGS cells after sihnRNPK treatment was analyzed by western blot. Unless otherwise specified, protein band intensity was normalized to β-tubulin band intensity. Significance Levels: *P < .05, ** *P* < .01, *** *P* < .001, ns: non-significant

To narrow down the candidates involved in post-transcriptional regulation of TPT1, we intersected the RNA-seq data with previously identified differentially expressed proteins from mass spectrometry analysis performed in HpSlyD-GFP stable cell lines [45]. Through the integrative approach, hnRNPK was identified as a potential key mediator of *H. pylori* SlyD-driven TPT1 regulation (Fig. 2A). HnRNPK is a well-known RNA-binding protein involved in the regulation of gene expression at the post-transcriptional level, influencing both mRNA stability and translation efficiency [27, 46, 47]. Subsequent validation experiments confirmed that hnRNPK was significantly upregulated in gastric epithelial cells following infection with *H. pylori* 26695, compared to the ΔSlyD strain and the blank group (Fig. 2B and C).

To further explore the functional role of hnRNPK in the regulation of TPT1, we performed hnRNPK knockdown experiments in *H. pylori* 26695-infected gastric epithelial cells. Interestingly, while TPT1 mRNA levels remained largely unchanged following hnRNPK knockdown, there was a marked decrease in TPT1 protein levels (Fig. 2D and E). In addition to the effects on TPT1 protein expression, hnRNPK knockdown also resulted in a significant decrease in the expression of GIM markers CDX2, Villin, and MUC2 (Fig. 2F and G). These results indicate that *H. pylori* SlyD upregulates hnRNPK expression, which in turn enhances TPT1 protein levels without affecting TPT1 mRNA transcription, contributing to the progression of GIM.

### *H. pylori* SlyD enhances TPT1 protein stability by promoting the interaction between hnRNPK and TPT1

**Fig. 3:**
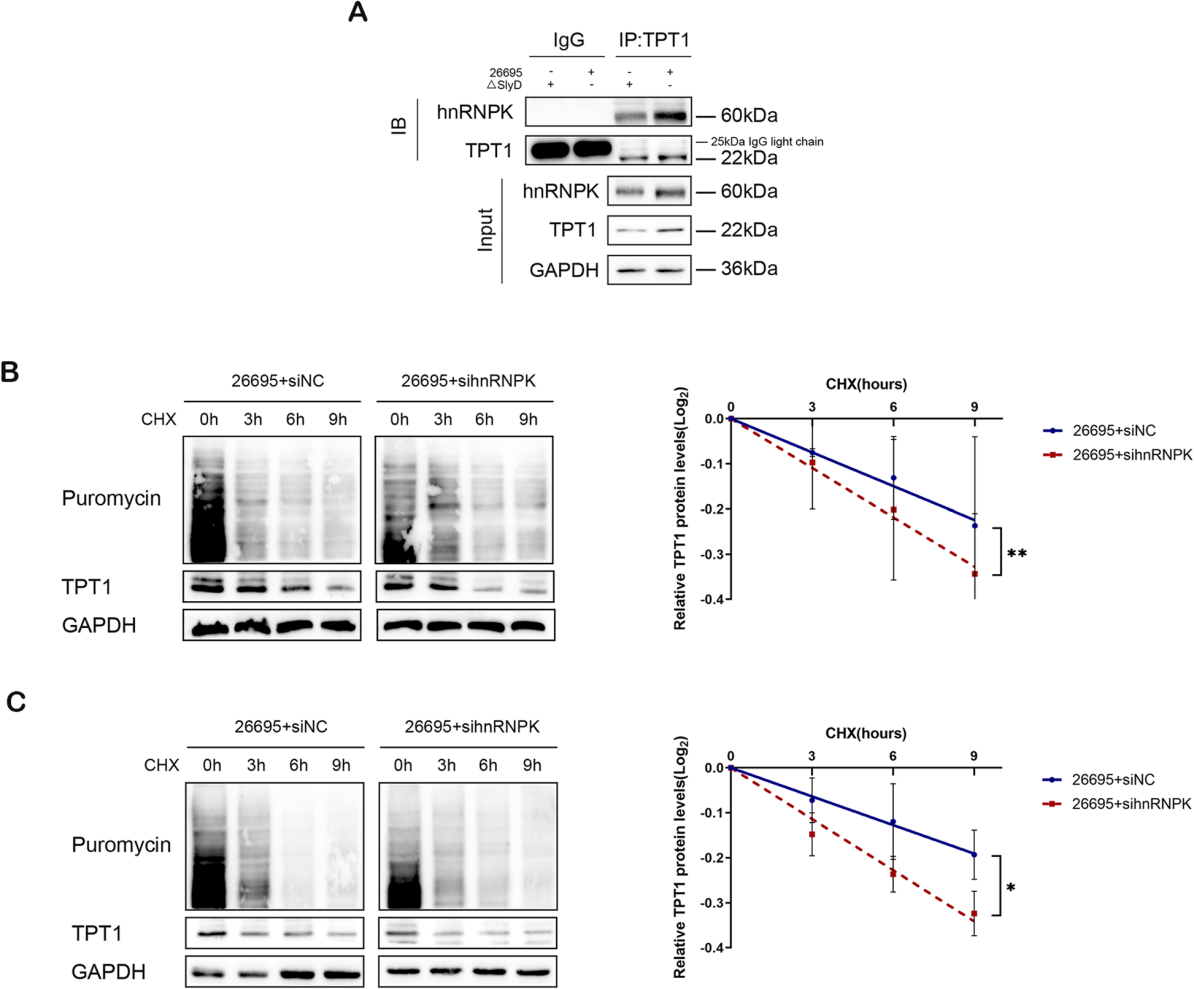
*H. pylori* SlyD enhances TPT1 protein stability by promoting the interaction between hnRNPK and TPT1. **A **Lysates from *H. pylori* SlyD-stimulated AGS were immunoprecipitated with IgG or anti-TPT1 antibody, followed by western blot to evaluate the expression levels of TPT1 and hnRNPK. **B **Western blot for TPT1 and puromycin expression after treated with CHX (80 μg/mL) and puromycin (10μg/mL) for different durations in *H. pylori* SlyD-stimulated GES-1 transfected with sihnRNPK or the control siRNA. **C **Western blot for TPT1 and puromycin expression after treated with CHX (80 μg/mL) and puromycin (10μg/mL) for different durations in *H. pylori* SlyD-stimulated AGS transfected with sihnRNPK or the control siRNA. GAPDH was used as internal loading control. Significance Levels: *P < .05, ** *P* < .01, *** *P* < .001, ns: non-significant

To gain further insight into the regulatory mechanisms by which hnRNPK influences TPT1 protein expression, we performed Co-IP assays to assess the interaction between hnRNPK and TPT1 in gastric epithelial cells. Our results revealed a direct interaction between hnRNPK and TPT1 (Fig. 3A). Notably, this interaction was significantly enhanced in cells infected with *H. pylori* 26695 compared to the ΔSlyD strain, indicating that the presence of *H. pylori* SlyD promotes the hnRNPK-TPT1 interaction.

Additionally, to explore the functional consequence of the hnRNPK-TPT1 interaction, we next examined the effect of hnRNPK on TPT1 protein stability. These assays were conducted in two gastric epithelial cell lines following hnRNPK knockdown. The results demonstrated that, under conditions of reduced nascent protein synthesis induced by CHX, reduced hnRNPK expression significantly shortened the half-life of TPT1 and accelerated its degradation (Fig. 3B and C). These results collectively suggest that *H. pylori* SlyD promotes the interaction between hnRNPK and TPT1, thereby increasing the stability of TPT1 protein in gastric epithelial cells.

### TPT1 promotes the expression of GIM markers by activating OCT1

**Fig. 4:**
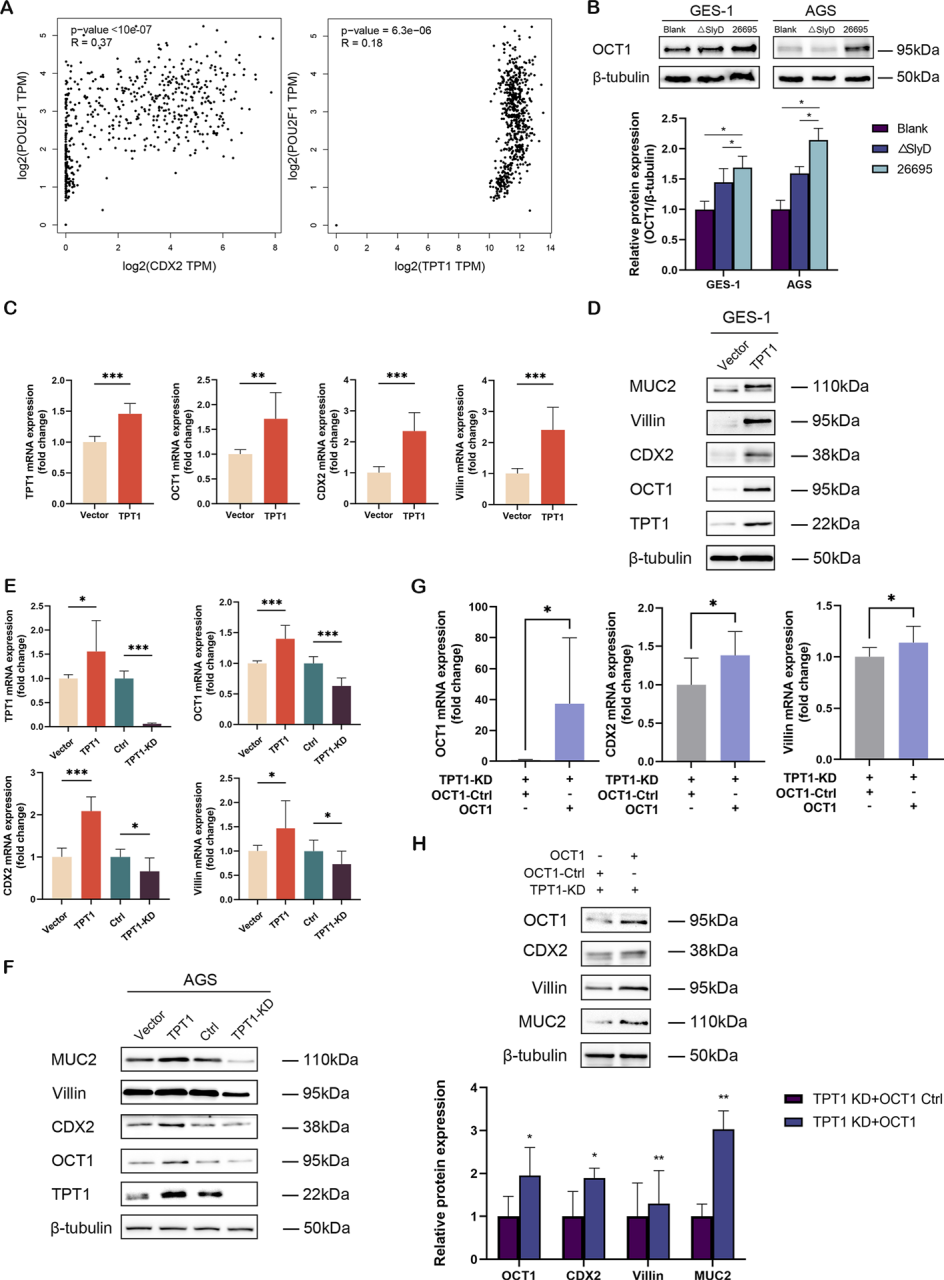
TPT1 promotes GIM marker expression by activating OCT1. **A** The GEPIA2.0 database was used to analyze the correlation between OCT1, TPT1 and CDX2 expression across various samples in TCGA and GTex databases. *R* is the correlation coefficient. **B **Western blot for OCT1 expression in *H. pylori* SlyD-stimulated GES-1 and AGS cells. **C **mRNA expression of OCT1, CDX2, and Villin in GES-1 cells overexpressing TPT1. **D **Western blot for the expression of OCT1 and GIM markers in GES-1 cells overexpressing TPT1. **E** mRNA expression levels of OCT1, CDX2, and Villin in AGS cells with overexpressed or downregulated TPT1 expression. **F **Western blot for OCT1 and GIM markers in AGS cells with overexpressed or downregulated TPT1 expression. **G **mRNA expression of GIM markers in stable OCT1-overexpressing cells with TPT1-knockdown. **H** Western blot for the expression of GIM markers in stable OCT1-overexpressing cells with TPT1-knockdown. β-tubulin was used as internal loading control. Significance Levels: *P < .05, ** *P* < .01, *** P < .001, ns: non-significant

To investigate how elevated TPT1 expression leads to the activation of the GIM driving factor CDX2, we utilized the UCSC genome browser to predict transcription factors that could potentially bind to the CDX2 promoter. Among the predicted candidates, members of the POU-domain transcription factor family were closely associated with CDX2 (Additional file 2: Fig S3). Next, we utilized the GEPIA 2.0 database to explore correlations between TPT1, CDX2, and specific POU-domain family members in gastric tissues. Our analysis revealed that both OCT1 and POU6F2 (RPF1), members of the POU family, showed positive correlations with TPT1 and CDX2 expression (Additional file 2: Fig S4). Importantly, OCT1 displayed a stronger correlation with both TPT1 and CDX2 than POU6F2 (Fig. 4A). And then we examined OCT1 levels in gastric epithelial cells infected with either *H. pylori* 26695 or ΔSlyD. The results show that *H. pylori* 26695 significantly upregulates OCT1 expression compared to ΔSlyD strain and the blank group (Fig. 4B).

To further establish the relationship between TPT1, OCT1, and GIM markers, we performed TPT1 overexpression experiments in GES-1 and AGS cell lines and found that overexpression of TPT1 resulted in a significant increase in OCT1 levels, as well as the upregulation of GIM markers (Fig. 4C–F). Additionally, overexpressed-OCT1 was able to rescue the reduction in GIM marker expression caused by TPT1 knockdown (Fig. 4G and H). This rescue effect highlights the critical role of OCT1 in mediating TPT1-driven GIM marker expression. Together, these results demonstrate that TPT1 promotes GIM markers via OCT1, with *H. pylori* SlyD serving as a key modulator in this pathway.

### OCT1 binds to the CDX2 promoter and directly regulates its transcription

**Fig. 5:**
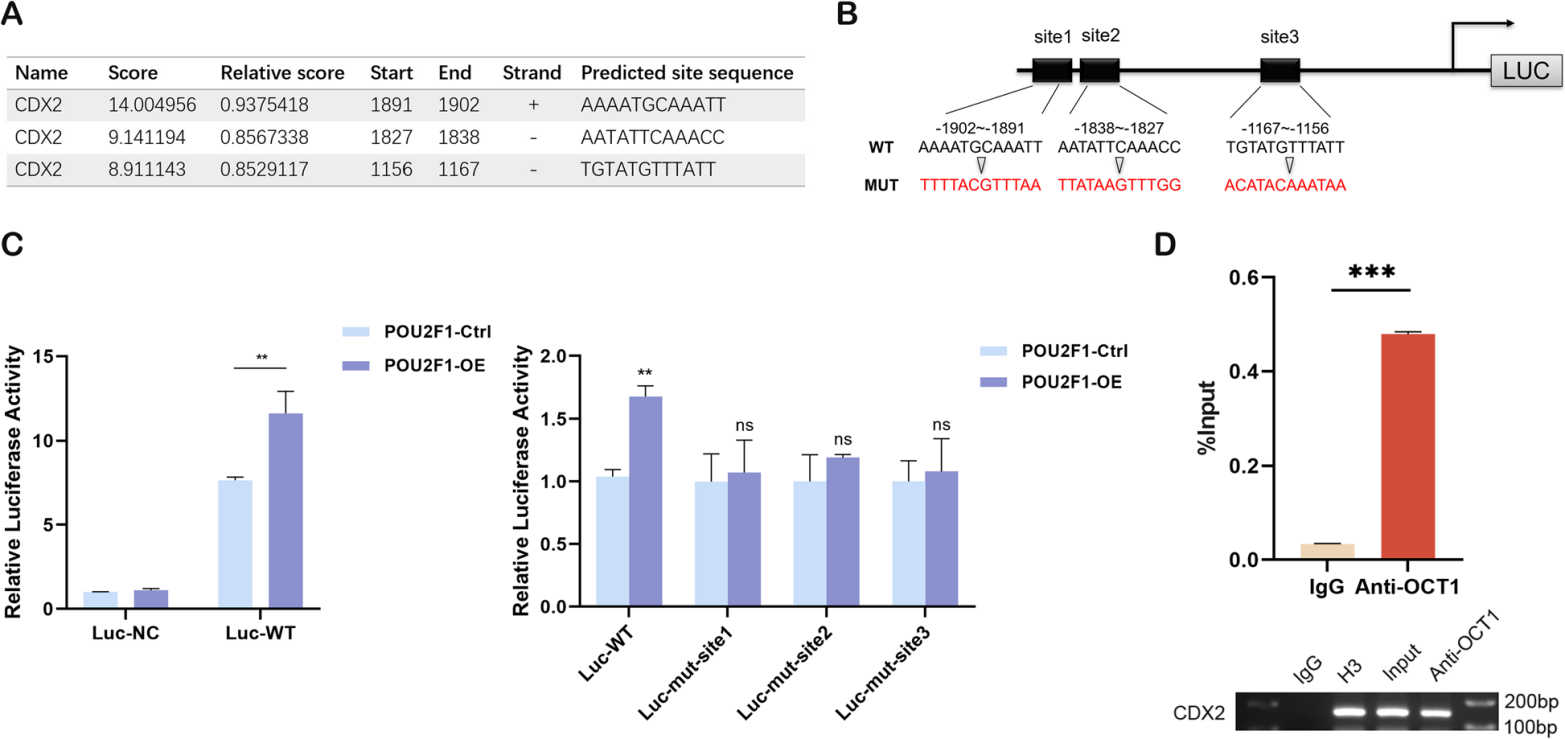
OCT1 binds to the CDX2 promoter and directly regulates its transcription. **A **Prediction of the binding sites of OCT1 to the promoter region of CDX2 by JASPAR database. **B **Potential binding sites and CDX2-promoter mutant constructs. **C **Dual-luciferase reporter assay to determine the relative luciferase activity of the CDX2 promoter (including wild-type and mutants). The luciferase assay was repeated for three times. **D **ChIP-qPCR to validate the binding of OCT1 to the CDX2 promoter region. The assay was repeated twice and represented as mean ± SD. Agarose gel electrophoresis showed the representative bands of two independent ChIP experiments. Significance Levels: *P < .05, **P < .01, ***P < .001, ns: non-significant

To elucidate the transcriptional regulatory mechanisms between OCT1 and CDX2, we employed the JASPAR database to predict potential binding sites of OCT1 within the CDX2 promoter region (Fig. 5A). A total of three potential binding sites were identified (Fig. 5B). And then dual-luciferase reporter assays were performed with transfecting constructs containing the CDX2 promoter region or its three mutants into HEK293 cells, followed by OCT1 overexpression. The results demonstrated a significant increase in luciferase activity at the CDX2 promoter when OCT1 was overexpressed compared to the negative control group. However, mutations in each of the three binding sites resulted in a notable reduction in luciferase activity (Fig. 5C). Next, we conducted ChIP-qPCR to further confirm the direct binding of OCT1 to the CDX2 promoter in vitro (Fig. 5D). These findings collectively indicate that OCT1 acts as a transcriptional regulator of CDX2, contributing to the upregulation of CDX2 expression.

### DHA targeting of TPT1 inhibits *H. pylori* SlyD-induced expression of GIM markers

**Fig. 6:**
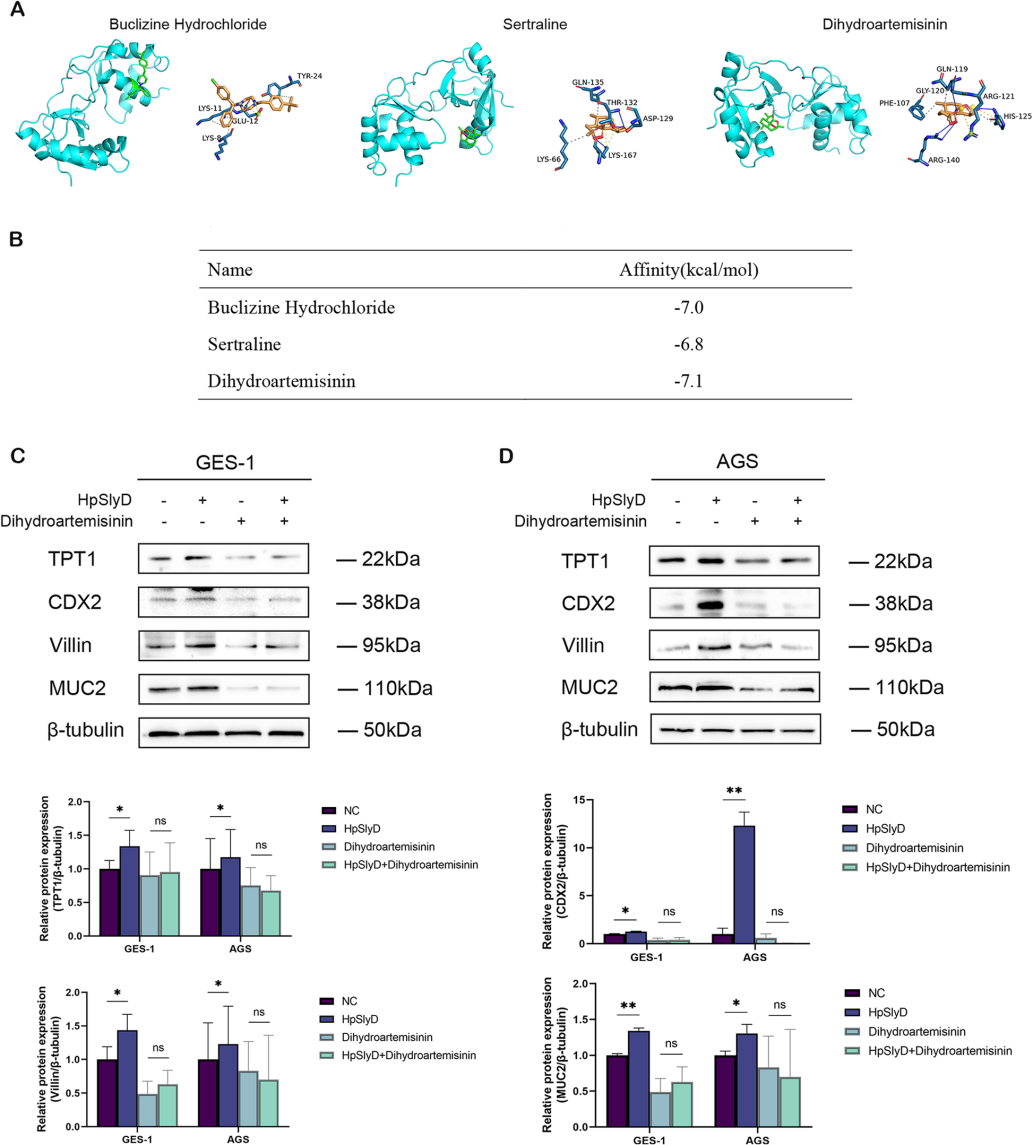
DHA targeting of TPT1 inhibits *H. pylori* SlyD-induced GIM marker expression. **A** Three-dimensional molecular structures and binding sites of buclizine hydrochloride, sertraline, and DHA when docked with TPT1. **B **The binding energy of buclizine hydrochloride, sertraline, and DHA when docked with TPT1. **C** Western blot for the expression of TPT1 and GIM markers in GES-1 cells treatment with purified HpSlyD proteins (200 ng/mL) for 40 h with or without DHA (10 μM). **D **Western blot for the expression of TPT1 and GIM markers in AGS cells treatment with purified HpSlyD proteins (200 ng/mL) for 40 h with or without DHA (10 μM). β-tubulin was used as internal loading control. Significance Levels: *P < .05, **P < .01, *** *P* < .001, ns: non-significant

Based on our findings highlighting the critical role of TPT1 in *H. pylori* SlyD-induced GIM marker expression, we sought to explore the potential clinical drugs targeting TPT1 to inhibit *H. pylori* SlyD-induced GIM. Previous reports had identified buclizine hydrochloride [48], sertraline [49], and DHA [50] as possible inhibitors of TPT1. Through molecular docking analyses, all three compounds were found to bind to TPT1 (identified interactions between molecules and |Affinity|> 5). Notably, the binding free energy between DHA and TPT1 was the highest, indicating robust interactions (Fig. 6A and B). Subsequently, supplementation of DHA to gastric epithelial cells followed by co-stimulation with purified HpSlyD protein revealed significant suppression of TPT1 levels, concomitant with downregulation of GIM marker expression (Fig. 6C and D). These results suggest that DHA, as a TPT1-targeting compound, holds promise as a potential inhibitor of *H. pylori*-related GIM marker expression.

### *H. pylori* SlyD is linked to hnRNPK/TPT1/OCT1/CDX2 expression in GIM of human gastric mucosa

**Fig. 7:**
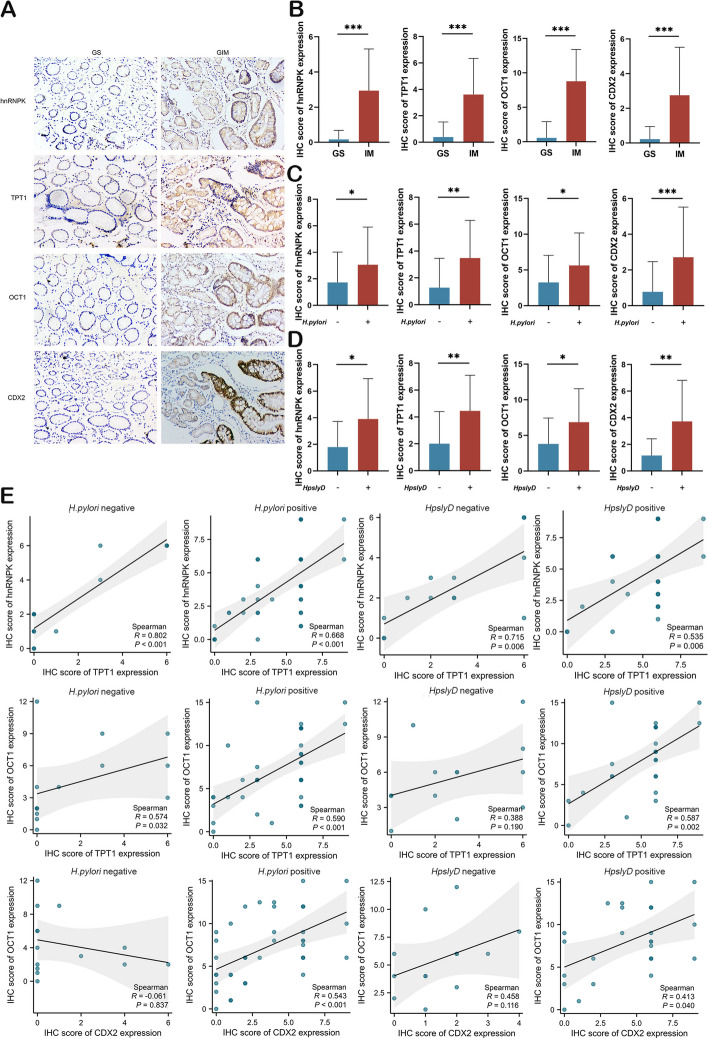
*H. pylori* SlyD is related to the expression of hnRNPK, TPT1, OCT1, and CDX2 in the IM of human gastric mucosa. **A **Representative images of hnRNPK/TPT1/OCT1/CDX2 IHC staining in GS and GIM, respectively (Magnification: ×100). **B** Comparison of semiquantitative scores of hnRNPK/TPT1/OCT1/CDX2 protein expression in GS (*n*=18) and GIM (*n*=52). **C** Comparison of semiquantitative scores of hnRNPK/TPT1/OCT1/CDX2 protein expression in *H. pylori*-negative (*n*=22) or *H. pylori*-positive (*n*=48) cases. **D **Comparison of semiquantitative scores of hnRNPK/TPT1/OCT1/CDX2 protein expression in *HpslyD*-negative (*n*=19) or *HpslyD*-positive (*n*=29) cases. **E **The scatter plots of hnRNPK and TPT1 expressions, OCT1 and TPT1 expressions, and OCT1 and CDX2 expressions, respectively in *H. pylori*-negative (*n*=14) or *H. pylori*-positive (*n*=38), and *HpslyD*-negative (*n*=13) or *HpslyD*-positive (*n*=25) cases. Significance Levels: *P< .05, **P< .01, ***P < .001, ns: non-significant

IHC staining was performed for hnRNPK, TPT1, OCT1, and CDX2 on 18 GS and 52 GIM tissues. Sample information and *H. pylori* subgroups are shown in Table 1. No statistical differences in gender and age were recorded between groups. HnRNPK, TPT1, OCT1, and CDX2 showed higher expression in GIM when compared to GS samples (Fig. 7A and B). *H. pylori*-positive patients exhibited elevated protein levels of hnRNPK, TPT1, OCT1, and CDX2 (Fig. 7C). Among *H. pylori*-positive patients, further stratification into *HpslyD*-negative and *HpslyD*-positive groups revealed increased protein expression of hnRNPK, TPT1, OCT1, and CDX2 in the *HpslyD*-positive group (Fig. 7D).

**Table 1:** The baseline information of human gastric tissue specimens used for IHC staining

Group	Characteristics	Total	*Hp* negative (%)	*Hp* positive (%)	*P*-value	*HpslyD* negative (%)	*HpslyD* positive (%)	*P*-value
**GS**	**Age**							
	** ≤ 60**	13	6(46.1)	7(53.9)	0.999	5(71.4)	2(28.6)	0.5
	** > 60**	5	2(40)	3(60)		1(33.3)	2(66.7)	
	**Gender**							
	**Female**	10	5(50)	5(50)	0.664	4(80)	1(20)	0.524
	**Male**	8	3(37.5)	5(62.5)		2(40)	3(60)	
	**Total**	18	8(44.4)	10(55.6)		6(60)	4(40)	
**GIM**	**Age**							
	** ≤ 60**	34	9(26.5)	25(73.5)	0.999	10(40)	15(60)	0.473
	** > 60**	18	5(27.8)	13(72.2)		3(23.1)	10(76.9)	
	**Gender**							
	**Female**	24	7(29.2)	17(70.9)	0.736	4(23.5)	13(76.5)	0.307
	**Male**	28	7(25)	21(75)		9(42.9)	12(57.1)	
	**Total**	52	14(26.9)	38(73.1)		13(34.2)	25(65.8)

And then we examined the correlations between hnRNPK, TPT1, OCT1, and CDX2 in GIM samples across different *H. pylori* subgroups. Results indicated a positive correlation between OCT1 expression and TPT1/CDX2 levels in *H. pylori*-positive and *HpslyD*-positive groups, with no significant associations in *H. pylori*-negative and *HpslyD*-negative groups (Fig. 7E). Notably, hnRNPK expression positively correlated with TPT1 irrespective of *H. pylori* infection status. These findings suggest an association between *H. pylori* SlyD and the expression of hnRNPK/TPT1/OCT1/CDX2 in GIM in human gastric mucosa, highlighting the potential role of these molecules contribute to the development of GIM during *H. pylori* infection.

### *H. pylori* SlyD is related to hnRNPK/TPT1/OCT1/CDX2 expression in GIM of MGs gastric mucosa

**Fig. 8:**
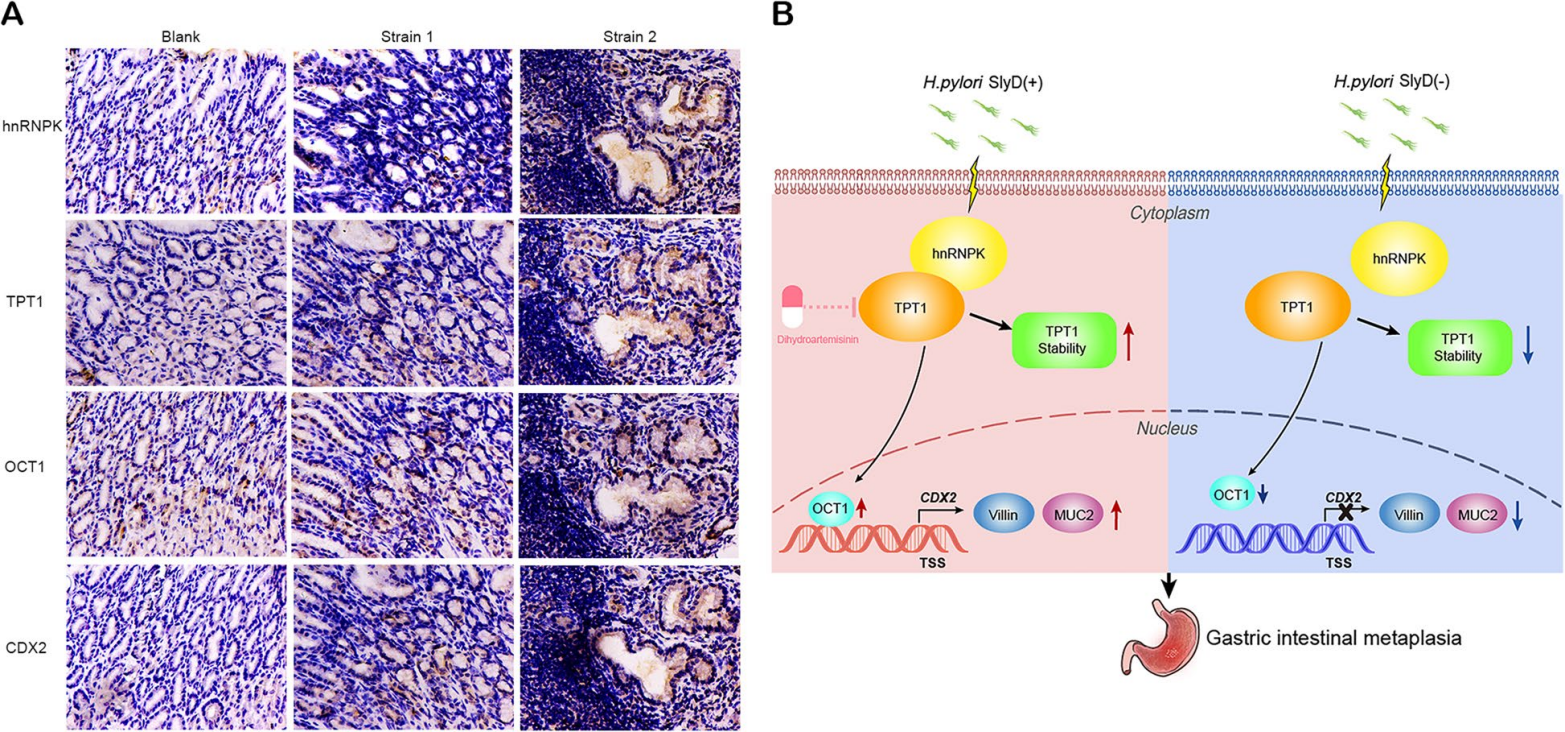
*H. pylori* SlyD induces GIM by stabilizing hnRNPK, upregulating TPT1, and enhancing OCT1-mediated CDX2 transcription. **A **IHC staining images of MGs gastric mucosa were presented (Magnification: ×200) to visualize the expression of hnRNPK/TPT1/OCT1/CDX2 in the sham group, the gavage group of the *HpslyD*-negative strain (Strain 1), and *HpslyD*-positive strain (Strain 2). **B **The molecular mechanism by which *H. pylori *SlyD induces GIM in this study

MGs were orally administered with different *H. pylori* strains or saline, and their gastric tissues were collected after 73 weeks. Notably, significant inflammatory reactions were evident in the gastric mucosa of mice infected with strain 2, accompanied by the presence of GIM glands (Fig. 8A). IHC staining of the strain 2-infected group revealed heightened expression of hnRNPK, TPT1, OCT1, and CDX2 in the gastric mucosa compared to the blank and strain 1 group (Fig. 8A). These findings indicate that *H. pylori* SlyD induced inflammation and GIM in mice, potentially linked to increased expression of hnRNPK, TPT1, OCT1, and CDX2.

## Discussion

GIM, recognized as a premalignant stomach lesion, markedly elevates the risk of GC development [51]. Our investigation revealed the crucial involvement of SlyD, an *H. pylori* virulence factor, in the progression of GIM. Specifically, *H. pylori* SlyD played essential roles in enhancing TPT1 stability through hnRNPK and facilitating OCT1-mediated transcriptional activation of CDX2. Moreover, targeting TPT1 with DHA effectively inhibited the upregulation of GIM marker expression induced by *H. pylori* SlyD (Fig. 8B).

Our research demonstrated that *H. pylori* SlyD upregulates TPT1 protein expression without altering its mRNA level. This upregulation of TPT1 protein correlates with increased expression of GIM markers, suggesting that *H. pylori* SlyD affects GIM through a potential post-transcriptional regulation of TPT1. Regulation of TPT1 can occur at various stages, including transcriptional, post-transcriptional, and post-translational processes. For instance, the tumor suppressor p53 is known to transcriptionally represses TPT1 expression [52–54], while the TPT1 protein undergoes modifications such as acetylation, ubiquitination, and post-translationally regulation by RNA-binding proteins [55, 56]. In our efforts to elucidate how *H. pylori* SlyD post-translational regulates TPT1, we employed RNA-seq data, mass spectrometry data, and in vitro experiments to investigate the interplay between TPT1 and *H. pylori* SlyD. Additionally, we found that *H. pylori* SlyD can promote the protein expressions of TPT1 and GIM markers by upregulating hnRNPK. HnRNPK, functioning as an RNA-binding protein, typically interacts with target RNAs to regulate transcription, RNA splicing, and translation processes. Characterized by a high content of proline residues and a KH-Domain Poly(C), hnRNPK also engages in protein–protein interactions with various functional domains to influence the expression levels of associated proteins [57, 58]. The existing studies have not reported the regulation of TPT1 by hnRNPK, so the regulatory relationships between hnRNPK and TPT1 remain unclear. Our study revealed that hnRNPK enhances the protein stability of TPT1 in gastric epithelial cells. Notably, hnRNPK and TPT1 form a protein–protein interaction, which is significantly enhanced by *H. pylori* SlyD, thereby increasing the expression of TPT1 and GIM markers. Additionally, IHC staining of human and MGs gastric tissues revealed a correlation between hnRNPK, TPT1, GIM, and *H. pylori* SlyD infection. These results provide new molecular insights into the mechanisms by which *H. pylori* SlyD regulates TPT1 expression.

So how does TPT1 promote GIM marker expression? We discovered that TPT1 promotes the expression of GIM markers through OCT1, while *H. pylori* SlyD plays an important role in this regulatory pathway. OCT1 is a key member of POU family, plays an essential role in various malignant diseases alongside other POU family members [59–61]. Studies have shown that TPT1 can interact with other POU family members such as POU5F1 (OCT4), but the relationship between TPT1 and OCT1 has not been reported [62–65]. Unfortunately, we did not detect direct interactions between TPT1 and OCT1 proteins in gastric epithelial cells (data not shown), so we postulate that TPT1 may indirectly regulate OCT1 through intermediary molecules. For example, both TPT1 [66, 67] and OCT1 [68, 69] are involved in regulating PI3K/AKT and AMPK/mTOR signaling pathways, suggesting a potential mechanism through which TPT1 may impact OCT1 activity. Further investigation is warranted to discover the precise regulatory interactions between TPT1 and OCT1, in the context of GIM development facilitated by *H. pylori* infection.

In our study, we found that OCT1 binds to the CDX2 promoter and upregulates CDX2 through transcriptional activation of its promoter. The activation of CDX2 has been identified as a crucial event in the initiation of GIM [1, 15, 70]. As a driving factor of GIM, CDX2 is regulated by various factors including Wnt/β-catenin [71], TGF-β [72], and miRNAs [73]. Previous studies indicated the high expression levels of OCT1 in GIM and GC tissues, and its ability to bind to the CDX2 promoter has been recognized [34, 74–76]. However, the ability of OCT1 to transcriptionally activate CDX2 remains controversial [34, 76]. Our study offers new evidence by identifying three binding sites on CDX2 promoter for OCT1 by dual-luciferase reporter assays. We demonstrated that OCT1 enhances the transcriptional activity of the CDX2 promoter through these binding sites, thereby upregulating CDX2 expression and promoting the development of GIM. Additionally, our analyses of human and mouse gastric tissues confirmed elevated expression levels, and a positive correlation among TPT1, OCT1, and CDX2 in GIM which are associated with *H. pylori* SlyD infection.

Our study highlights the significant role of TPT1 in *H. pylori* SlyD-induced GIM and explores the potential of targeting TPT1 to block the expression of GIM markers. Continuously, we discovered that DHA, a compound targeting TPT1, is expected to be a potential inhibitor of *H. pylori*-associated GIM. Sertraline [49, 77], buclizine hydrochloride [48], and DHA [50, 78] have been identified as possible inhibitors of TPT1. Using molecular docking analysis, we found that DHA exhibited the strongest binding capacity to TPT1. In vitro experiments validated that DHA effectively inhibited TPT1 expression induced by *H. pylori* SlyD, leading to the downregulation of GIM markers. Previous research has shown that DHA not only inhibited *H. pylori*-induced GC [79] but also suppressed GC cell invasion, epithelial–mesenchymal transition [80, 81], and increased its chemosensitivity [82]. DHA has also demonstrated efficacy in suppressing differentiation in various cell lines associated with hematological [83], immunological [84], and urological [85] disorders, indicating its potential in delaying disease progression by reversing abnormal cell differentiation. These findings suggest that DHA, through its action on TPT1, may serve as a potential inhibitor of *H. pylori*-related gastric precancerous diseases. The inhibitory effects of DHA on GIM may provide new possibilities for its application in the management of gastric precancerous lesions.

There are still some deficiencies of our study that need to be improved. In this study, IHC staining determined that the expression of hnRNPK and TPT1 in GIM tissues was positively correlated irrespective of *H. pylori* infection status. Nevertheless, our studies have already confirmed the differential expressions of hnRNPK and TPT1 among different *H. pylori* subgroups in gastric epithelial cells, as well as in human and MGs gastric tissues. These findings confirm the facilitating roles of *H. pylori* SlyD on hnRNPK and TPT1 expressions in GIM are indisputable. Undeniably, expanding the sample size of IHC staining in future studies will be critical to further validate these results. Moreover, there are still some aspects that need to be explored more in the future. The lack of in vivo validations of GIM reversal through DHA treatment, and the differences of GIM phenotypes observed following the infection of *H. pylori* 26695 and ΔSlyD strains were mainly limited by the lengthy process required to establish animal models, which took at least 73 weeks. Although these deficiencies of animal experiments do not affect the accuracy of our conclusions, we will still focus on addressing these limitations in the future to further validate our observations.

## Conclusions

We demonstrated that *H. pylori* SlyD enhanced TPT1 protein stability through hnRNPK, facilitating OCT1-mediated transcriptional activation of CDX2 to trigger GIM. These results provide a deeper understanding of the molecular mechanisms underpinning gastric precancerous disease development, while DHA may be used for the early prevention and reversal of *H. pylori*-induced GIM.

## Supplementary Information

Additional file 1: Table S1-Primer and siRNA sequences. Table S2-Antibody information. Table S3-Genes upregulated by *H. pylori * SlyD identified through RNA-seq analysis. 

Additional file 2: Fig S1-GO enrichment analysis of genes upregulated in *H. pylori* SlyD-positive cell line. Fig S2-KEGG enrichment analysis of genes upregulated in *H. pylori* SlyD-positive cell line. Fig S3-Prediction of transcription factors potentially binding to the CDX2 promoter region by UCSC database. Fig S4-Analysis of the correlation between POU family members' expression and TPT1, CDX2 in gastric tissue using the GEPIA 2.0 database.

Additional file 3: Original images of blots and gels.

## Data Availability

The RNA-seq data is accessible at Gene Expression Omnibus (accession number: GSE282336). All remain data are included in the article and additional files, or available from the authors upon reasonable request.

## Abbreviations

3D: Three-dimensional
CDX2: Caudal type homeobox protein 2
CFU: Colony-forming units
ChIP: Chromatin immunoprecipitation
CHX: Cycloheximide
Co-IP: Co-immunoprecipitation
DEGs: Differentially expressed genes
DHA: Dihydroartemisinin
FBS: Fetal bovine serum
GC: Gastric cancer
GIM: Gastric intestinal metaplasia
*H.** pylori*: *Helicobacter pylori*
hnRNPK: Heterogeneous nuclear ribonucleoprotein K
IGC: Intestinal-type gastric cancer
IHC: Immunohistochemistry
IS: Immunohistochemical score
MGs: Mongolian gerbils
MOI: Multiplicity of infection
OCT1/POU2F1: Octamer-binding transcription factor 1/ POU Domain, Class 2, Transcription Factor 1
PDB: Program database file
PLIP: Protein-Ligand Interaction Profiler
RNA-seq: RNA sequencing
RPKM: Reads per Kilobase per Million Reads
RT-qPCR: Real-time quantitative PCR
SPF: Specific pathogen free
TPT1/TCTP: Translationally controlled tumor protein 1
ΔSlyD: SlyD inactivated mutant
