# Supplementary material for: Helicobacter pylori SlyD stabilizes TPT1 via hnRNPK and enhances OCT1-mediated CDX2 transcriptional activation to drive gastric intestinal metaplasia
Source: BMC Med. 2025 Feb 6;23:71. doi: 10.1186/s12916-025-03911-8 (PMC11803974; doi:10.1186/s12916-025-03911-8)
Supplement: Supplementary file 2 — Additional file 2: Fig S1-GO enrichment analysis of genes upregulated in H. pylori SlyD-positive cell line. Fig S2-KEGG enrichment analysis of genes upregulated in H. pylori SlyD-positive cell line. Fig S3-Prediction of transcription factors potentially binding to the CDX2 promoter region by UCSC database. Fig S4-Analysis of the correlation between POU family members' expression and TPT1, CDX2 in gastric tissue using the GEPIA 2.0 database. [file 12916_2025_3911_MOESM2_ESM.docx]

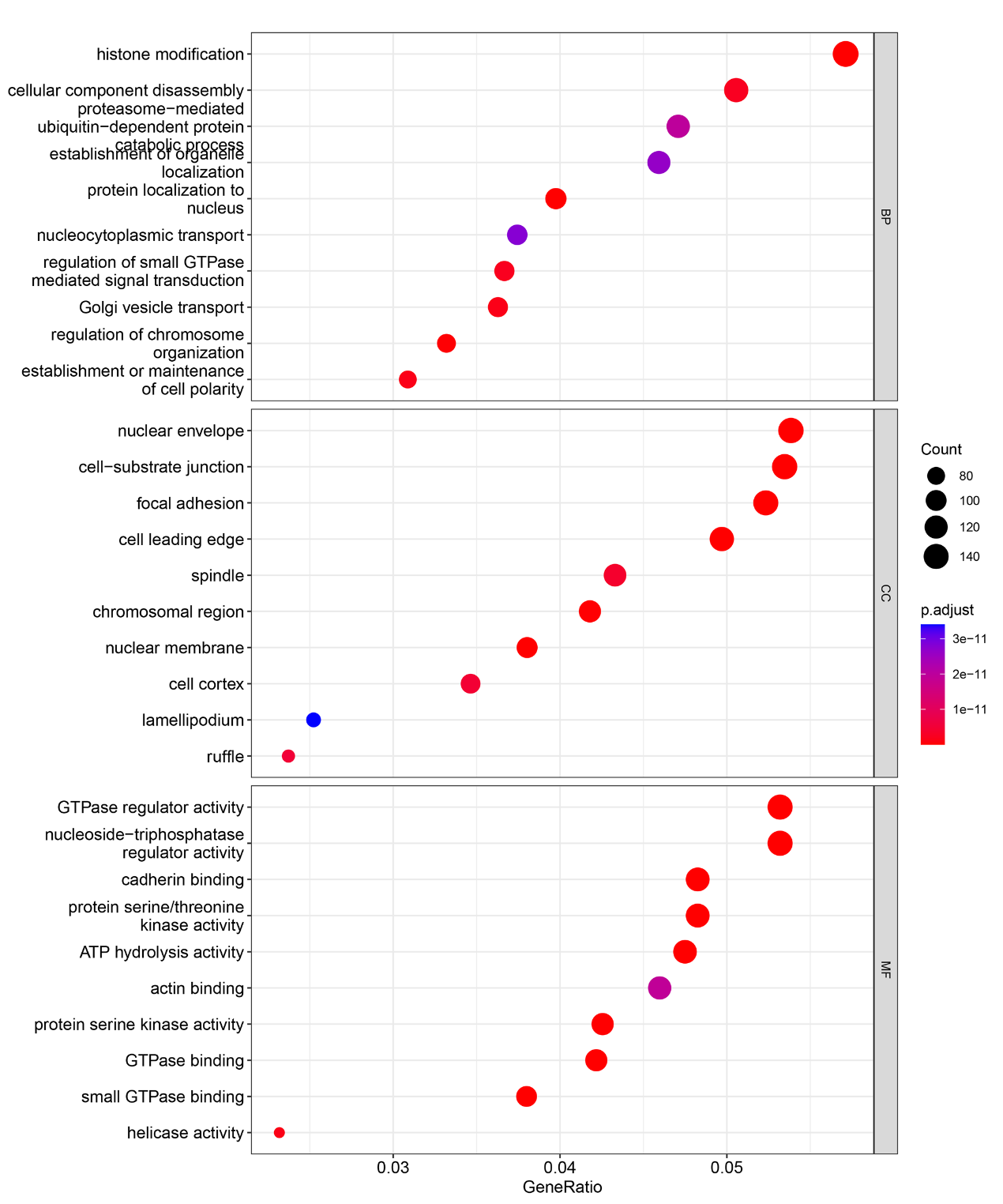


Fig S1. GO enrichment analysis of genes upregulated in *H.pylori* SlyD-positive cell line.


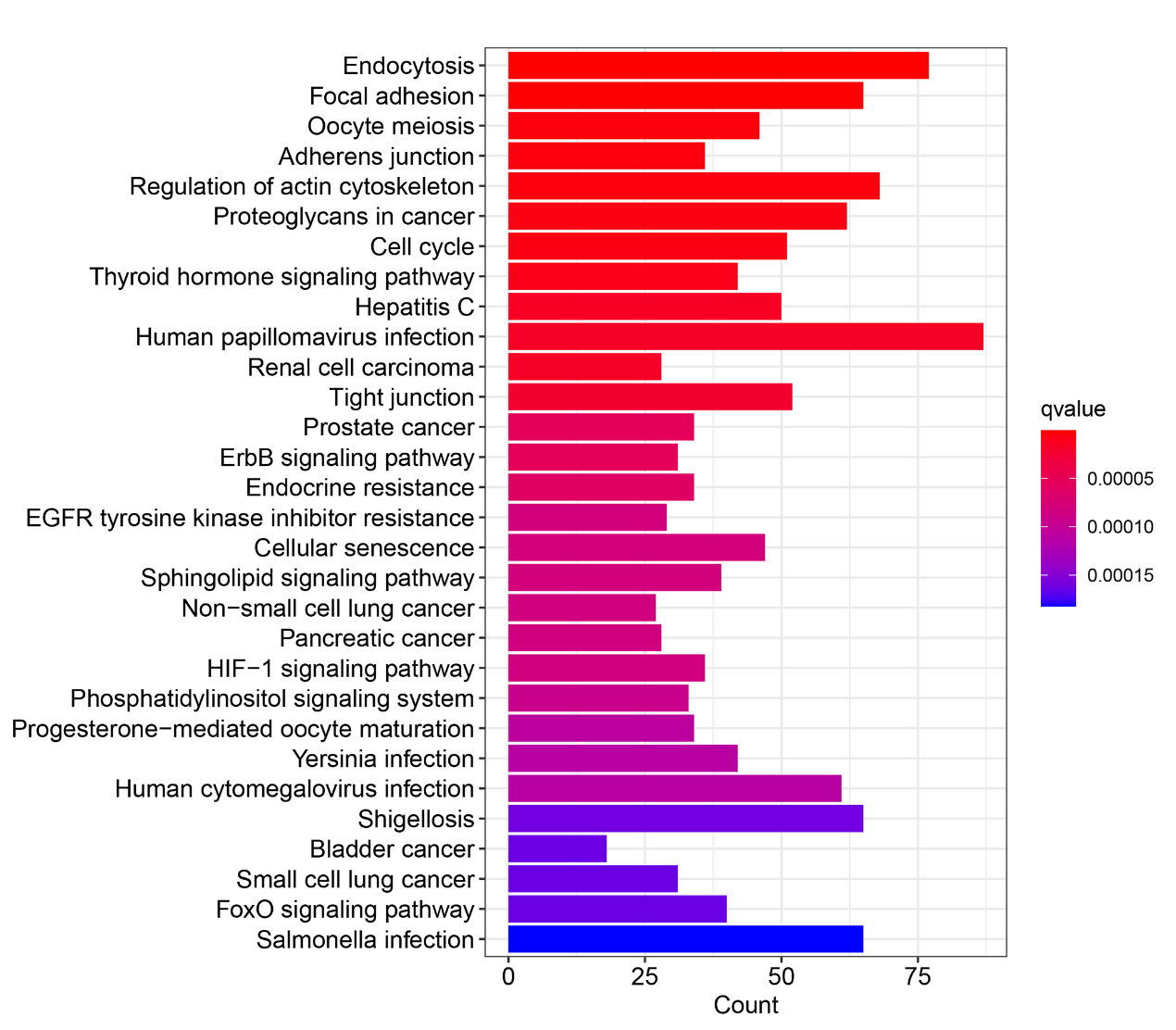


Fig S2. KEGG enrichment analysis of genes upregulated in *H.pylori* SlyD-positive cell line.


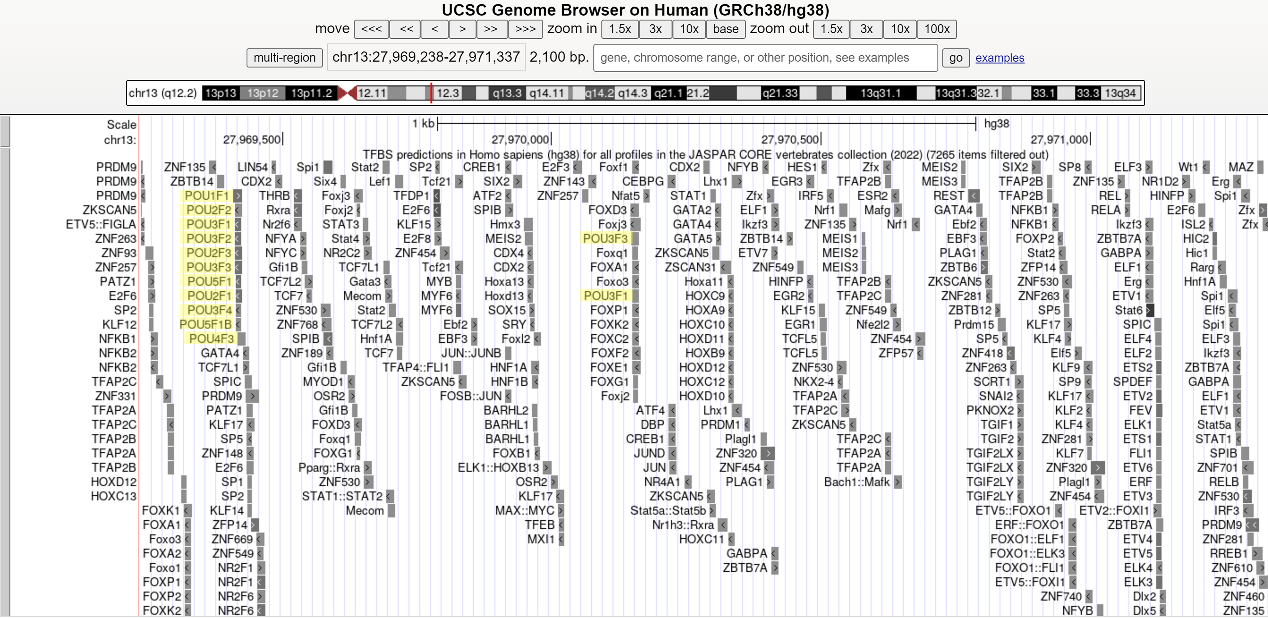


Fig S3. Prediction of transcription factors potentially binding to the CDX2 promoter region by UCSC database.


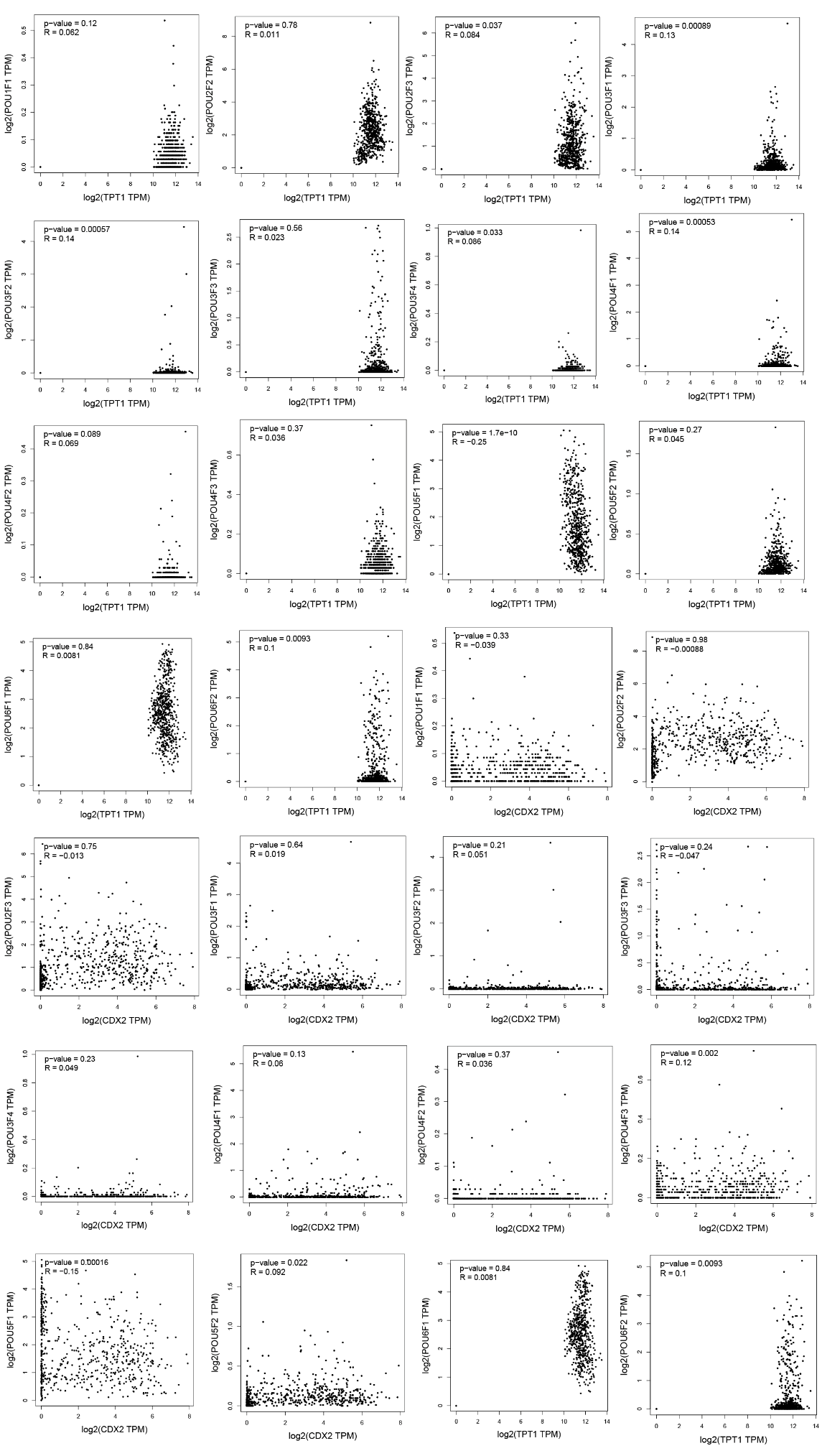


Fig S4. Analysis of the correlation between POU family members' expression and TPT1, CDX2 in gastric tissue using the GEPIA 2.0 database.
