# Supplementary material for: Helicobacter pylori SlyD stabilizes TPT1 via hnRNPK and enhances OCT1-mediated CDX2 transcriptional activation to drive gastric intestinal metaplasia
Source: BMC Med. 2025 Feb 6;23:71. doi: 10.1186/s12916-025-03911-8 (PMC11803974; doi:10.1186/s12916-025-03911-8)
Supplement: Supplementary file 3 — Additional file 3: Original images of blots and gels. [file 12916_2025_3911_MOESM3_ESM.docx]

**Original images of blots and gels**

**Figure 1B.**


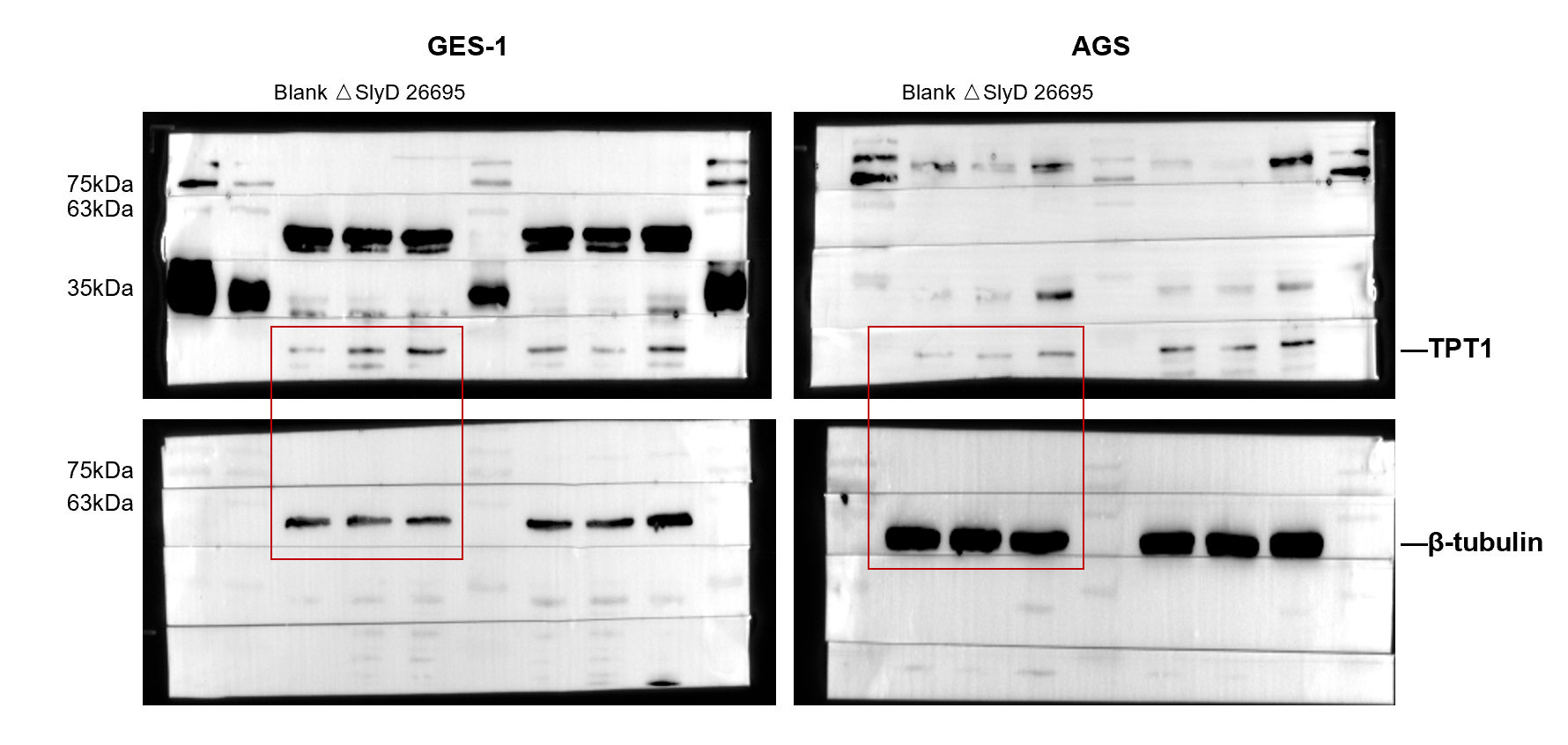


**Figure 1C.**


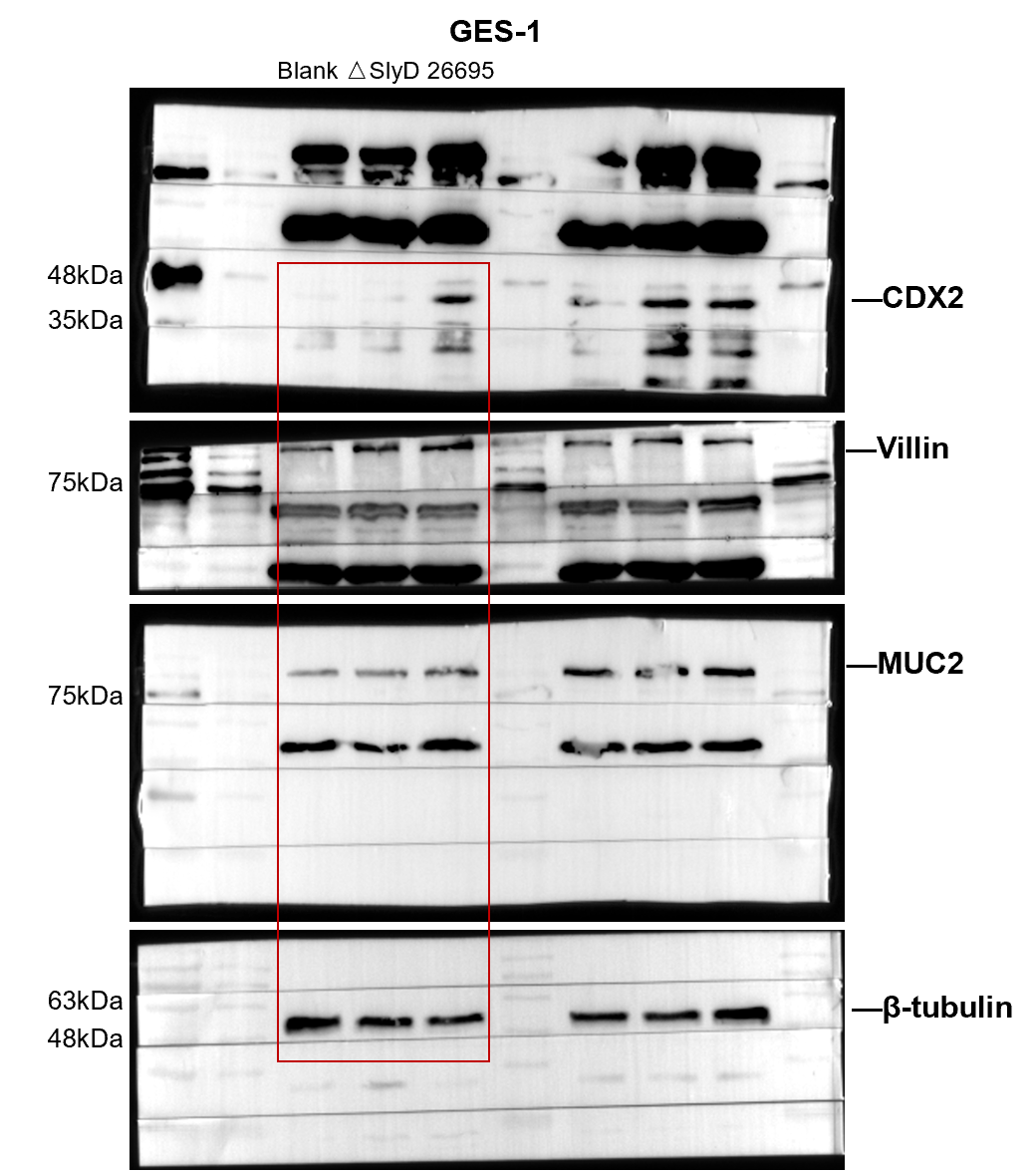


**Figure 1D.**

**
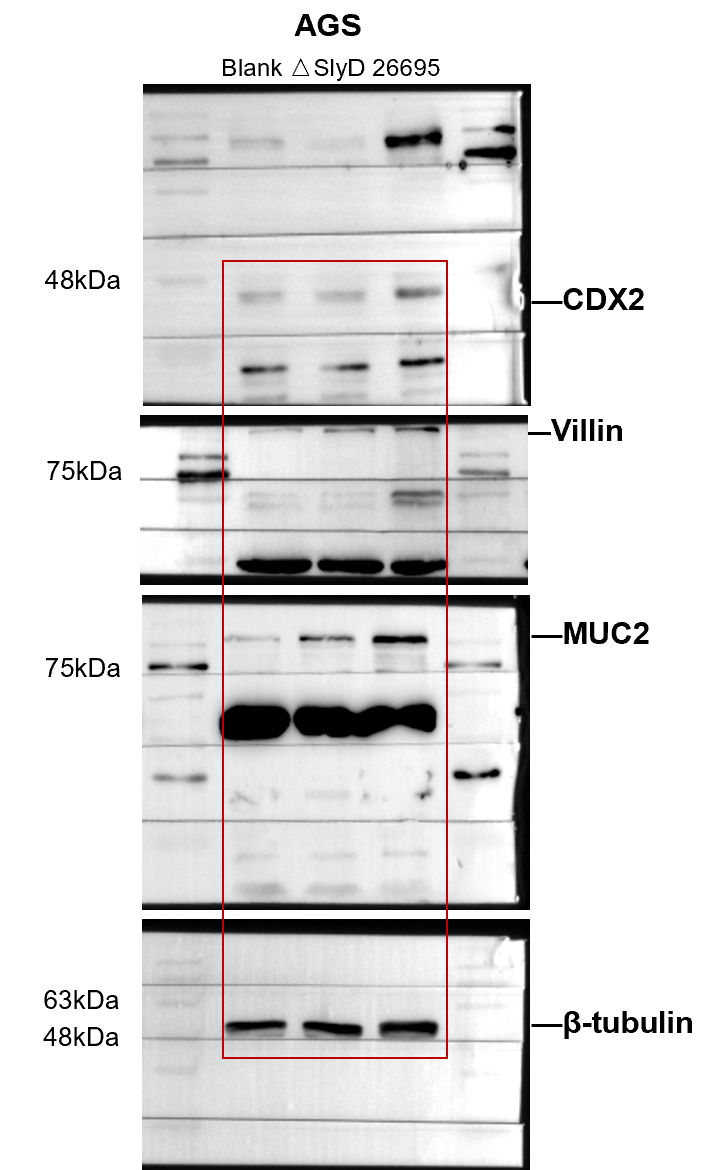
**

**Figure 1F.**

**
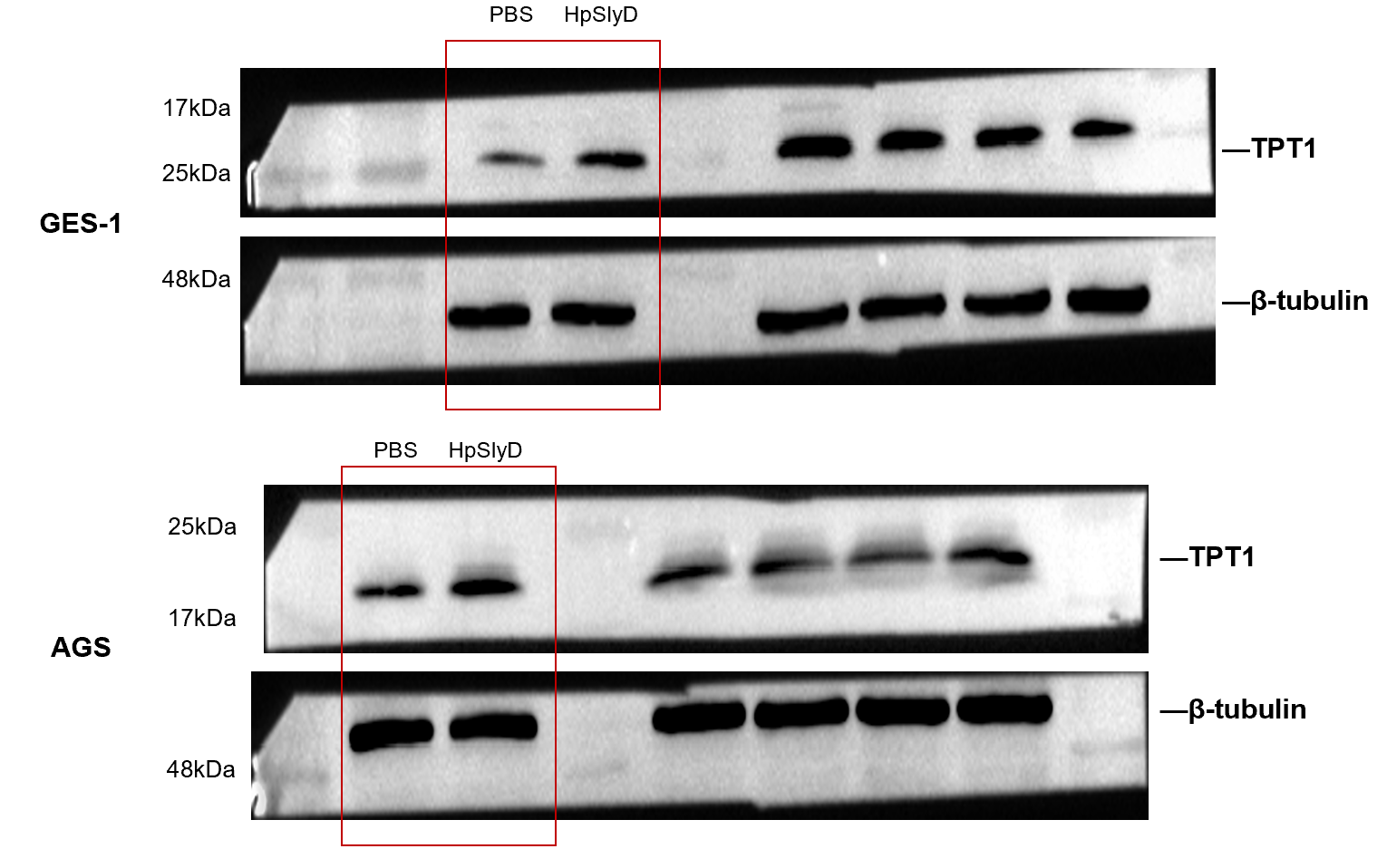
**

**Figure 1G.**

**
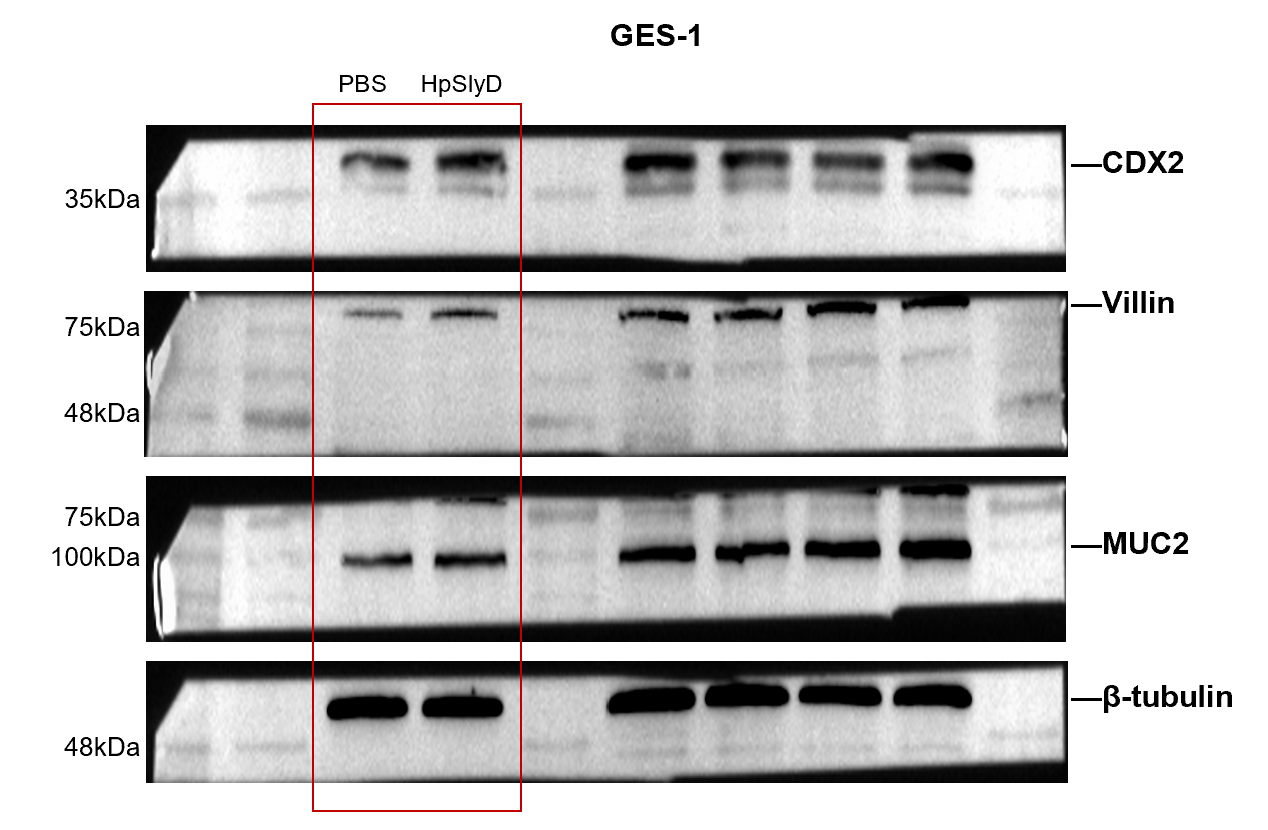
**

**Figure 1H.**

**
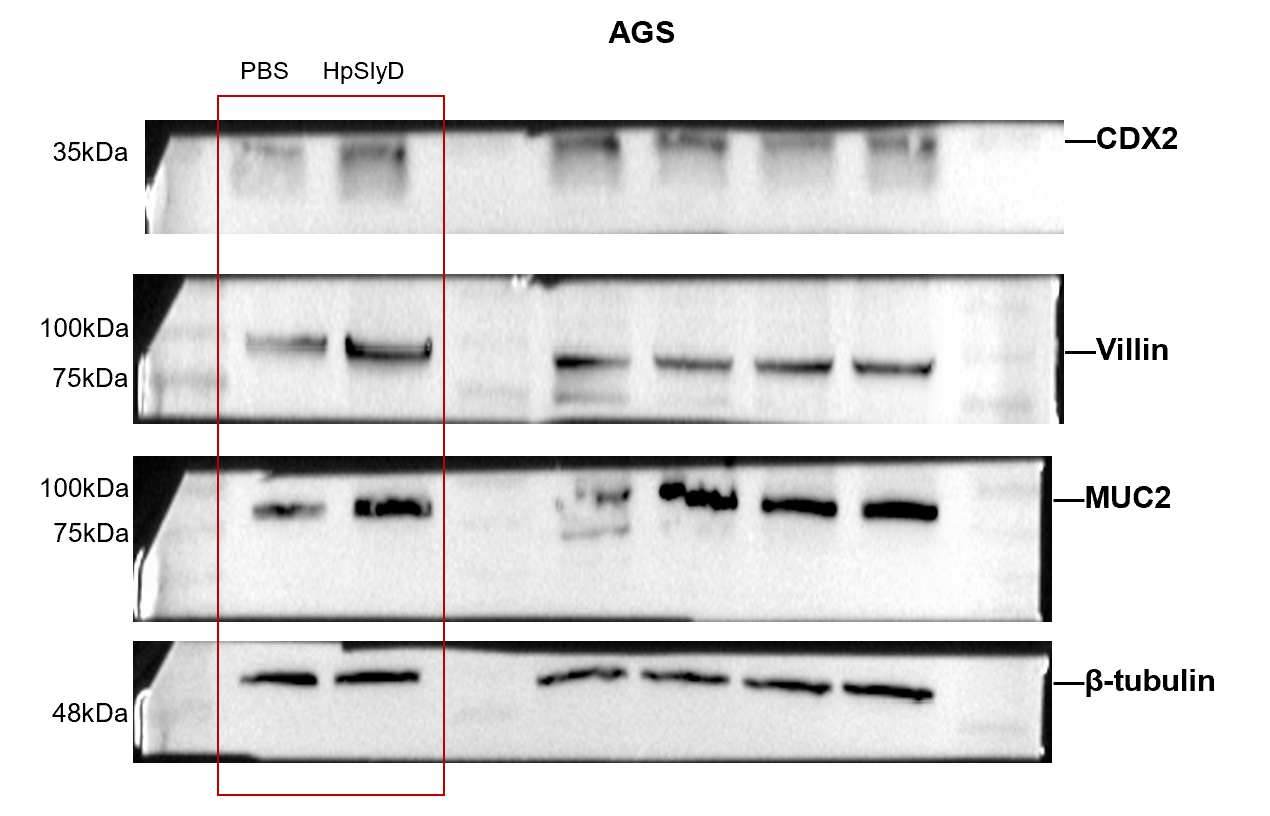
**

**Figure 2C.**

**
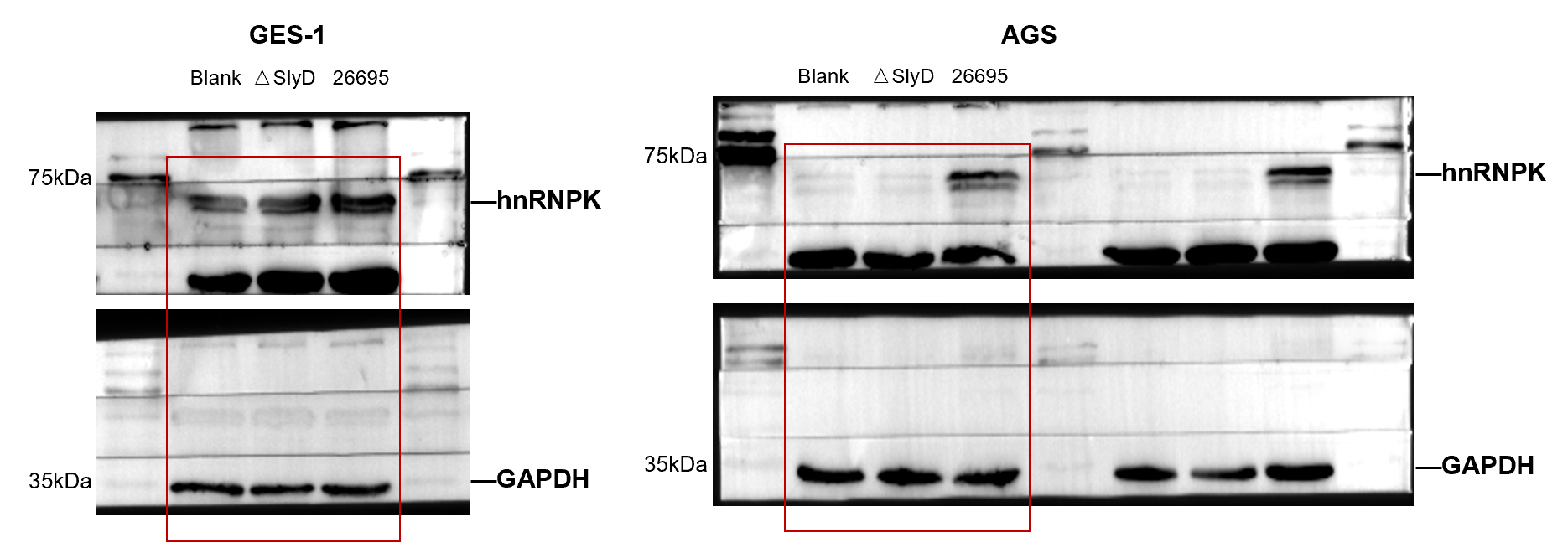
**

**Figure 2D.**

**
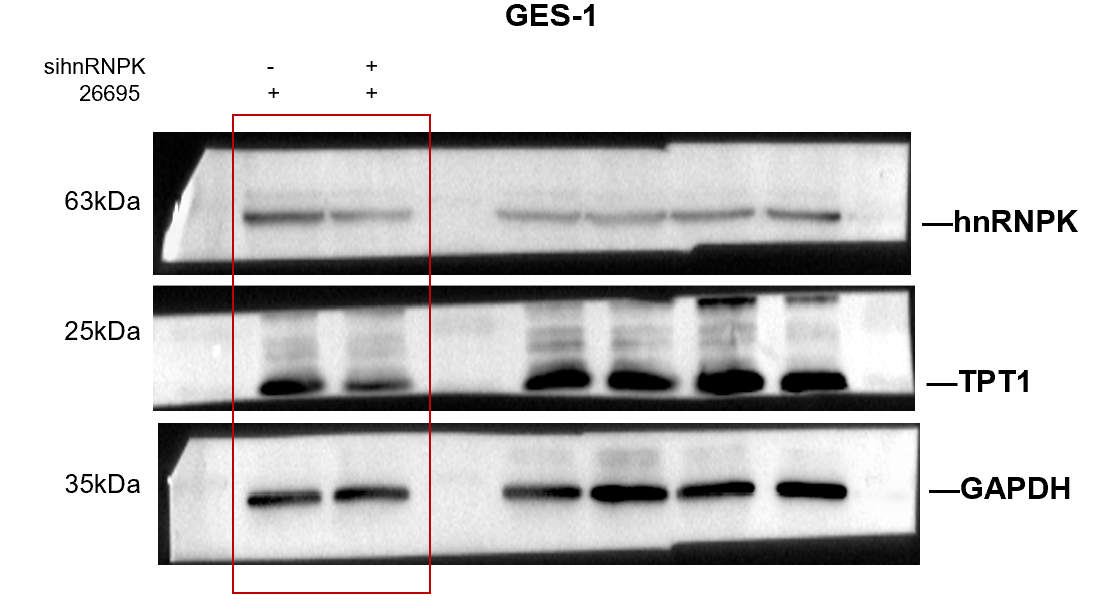
**

**Figure 2E.**

**
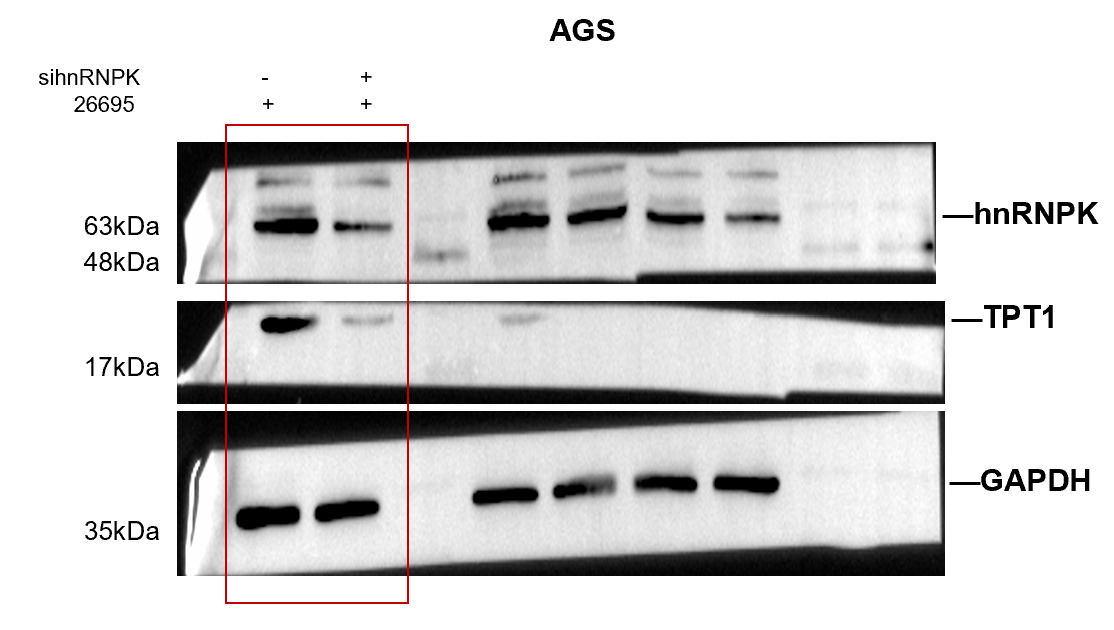
**

**Figure 2F.**

**
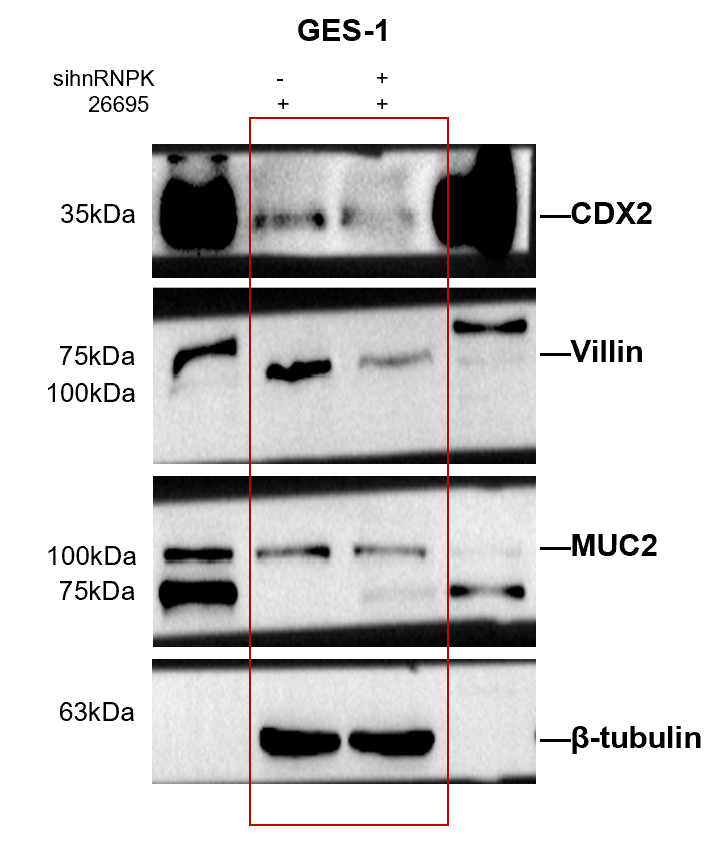
**

**Figure 2G.**

**
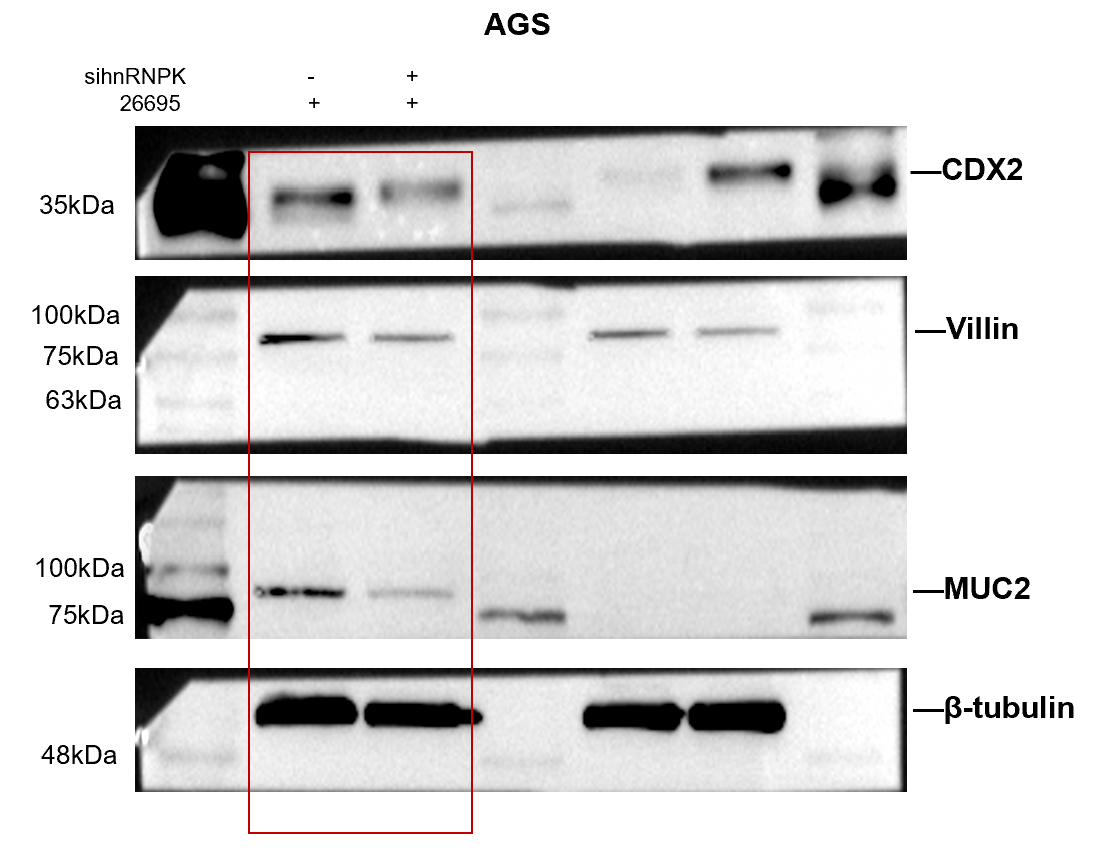
**

**Figure 3A.**

**
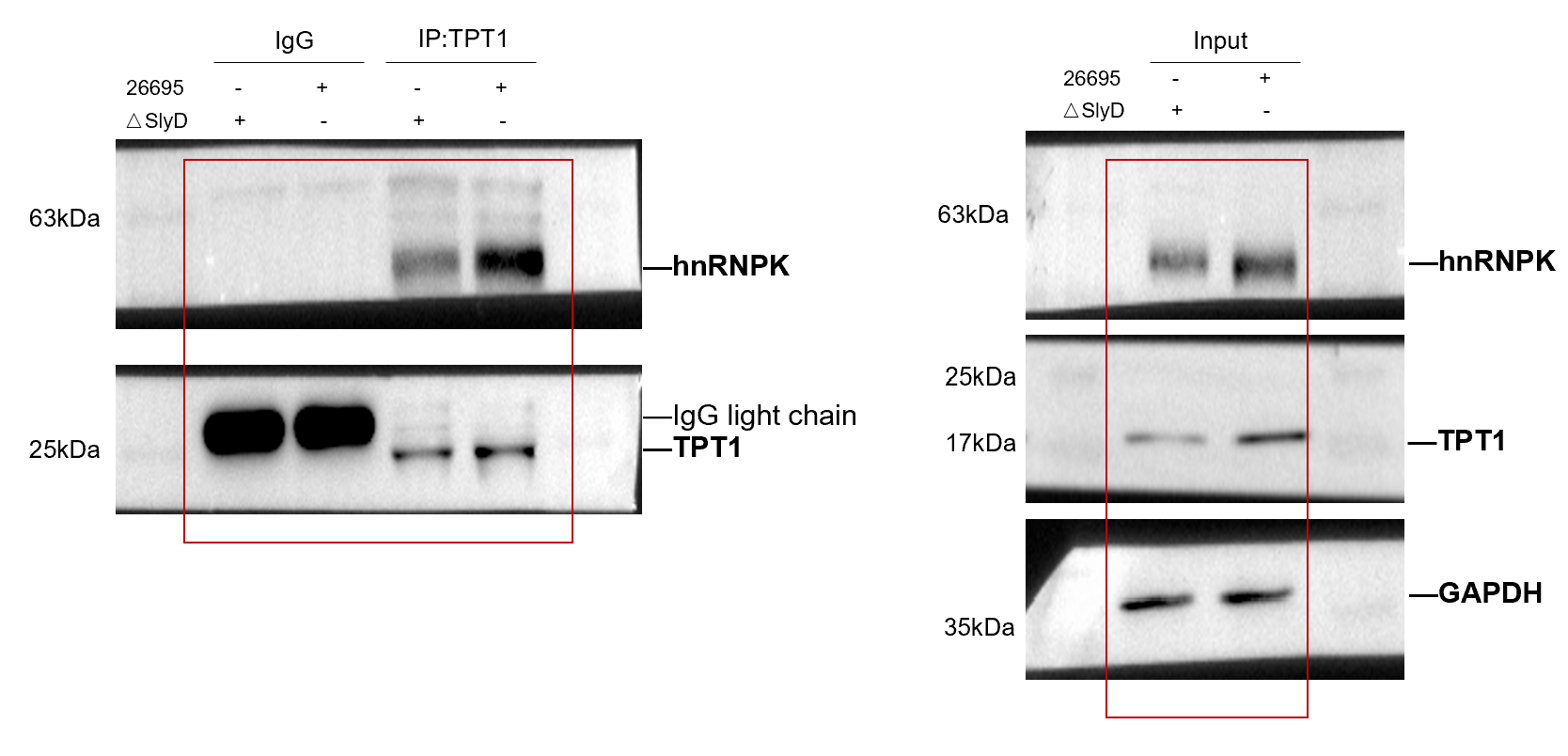
**

**Figure 3B.**

**
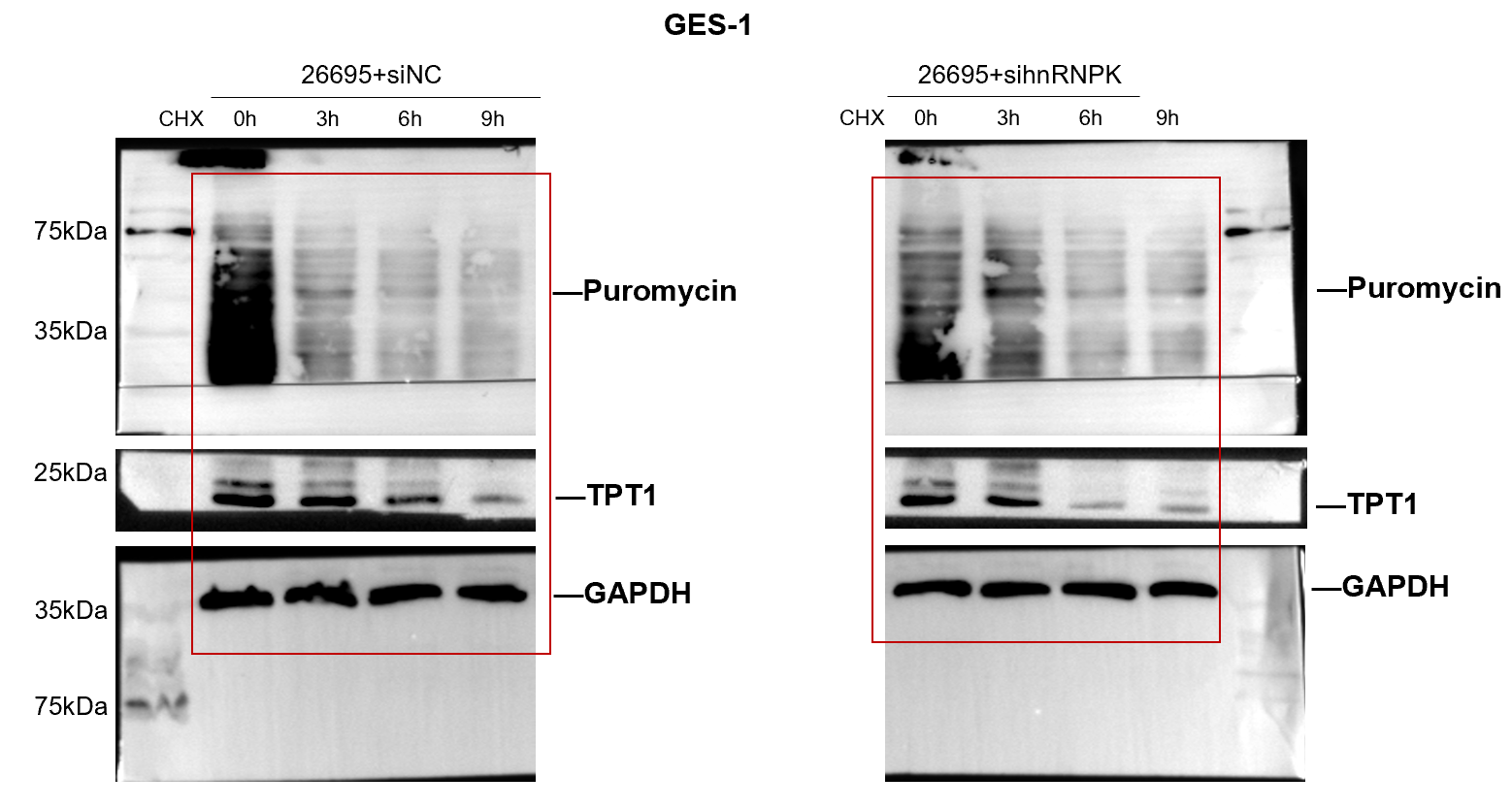
**

**Figure 3C.**

**
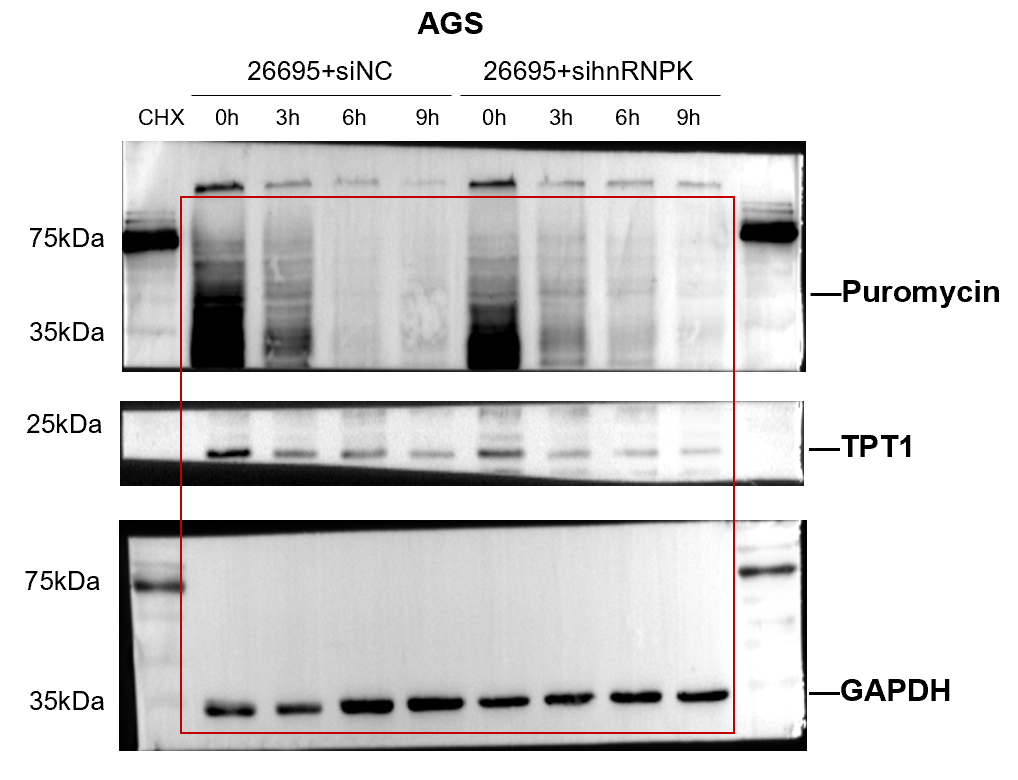
**

**Figure 4B.**

**
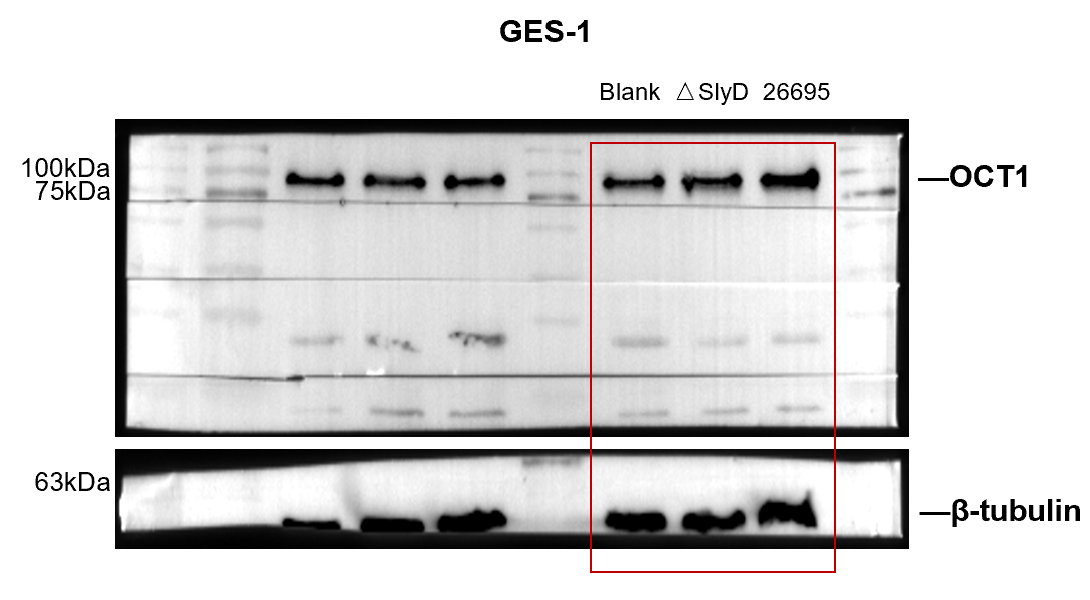
** **
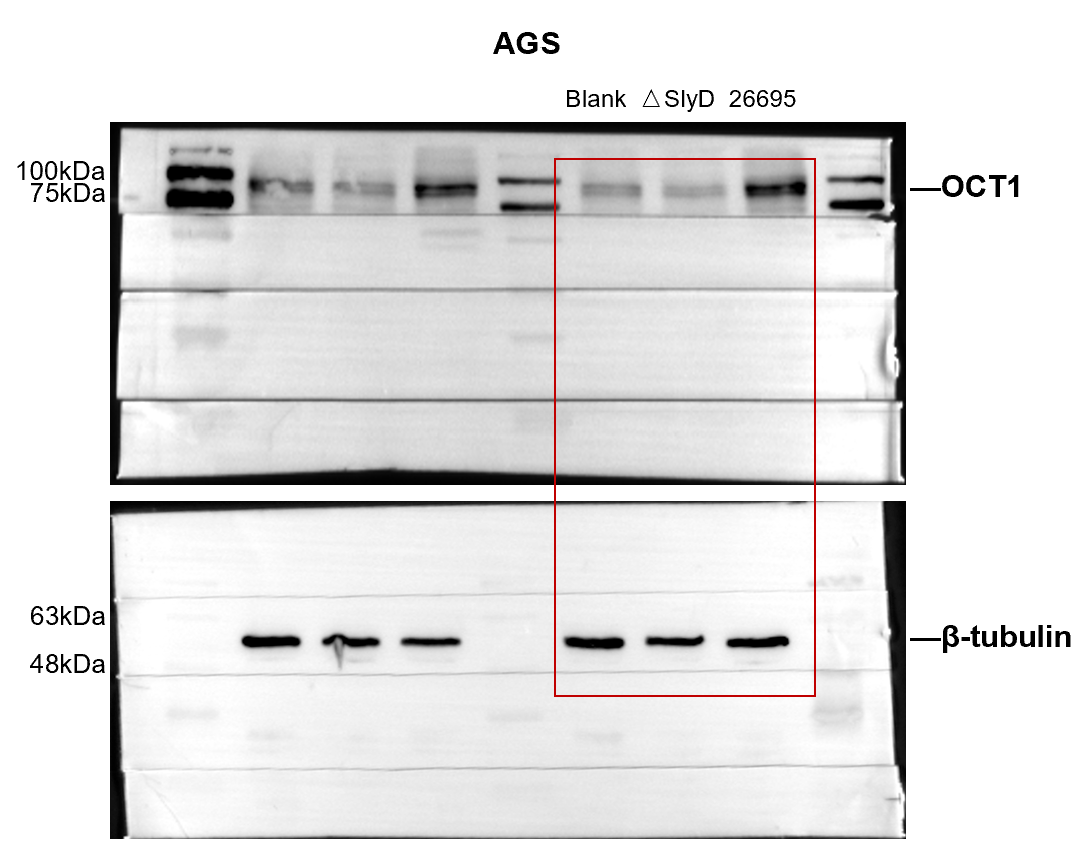
**

**Figure 4D.**

**
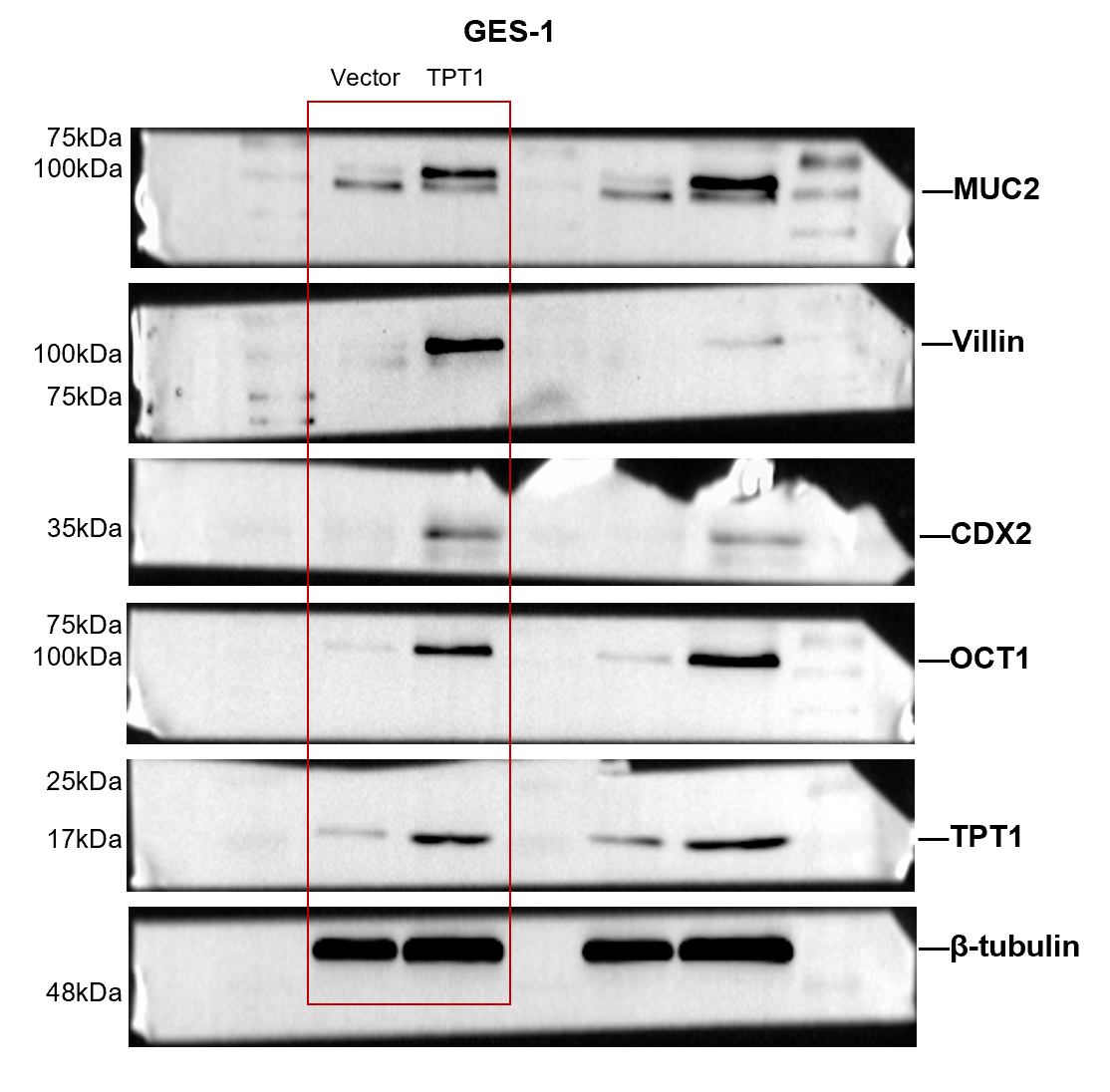
**

**Figure 4F.**

**
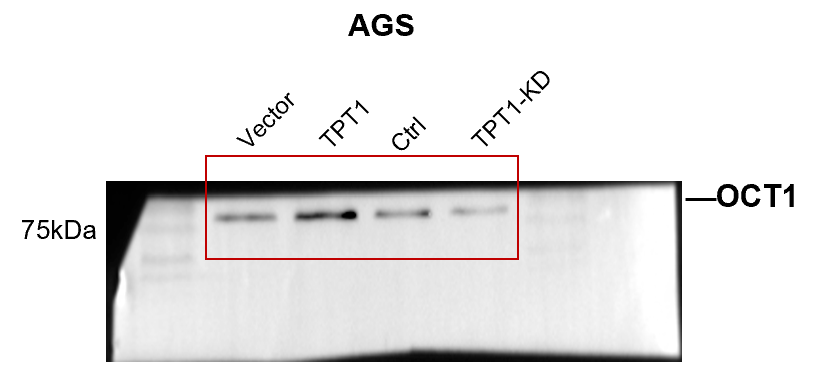
** **
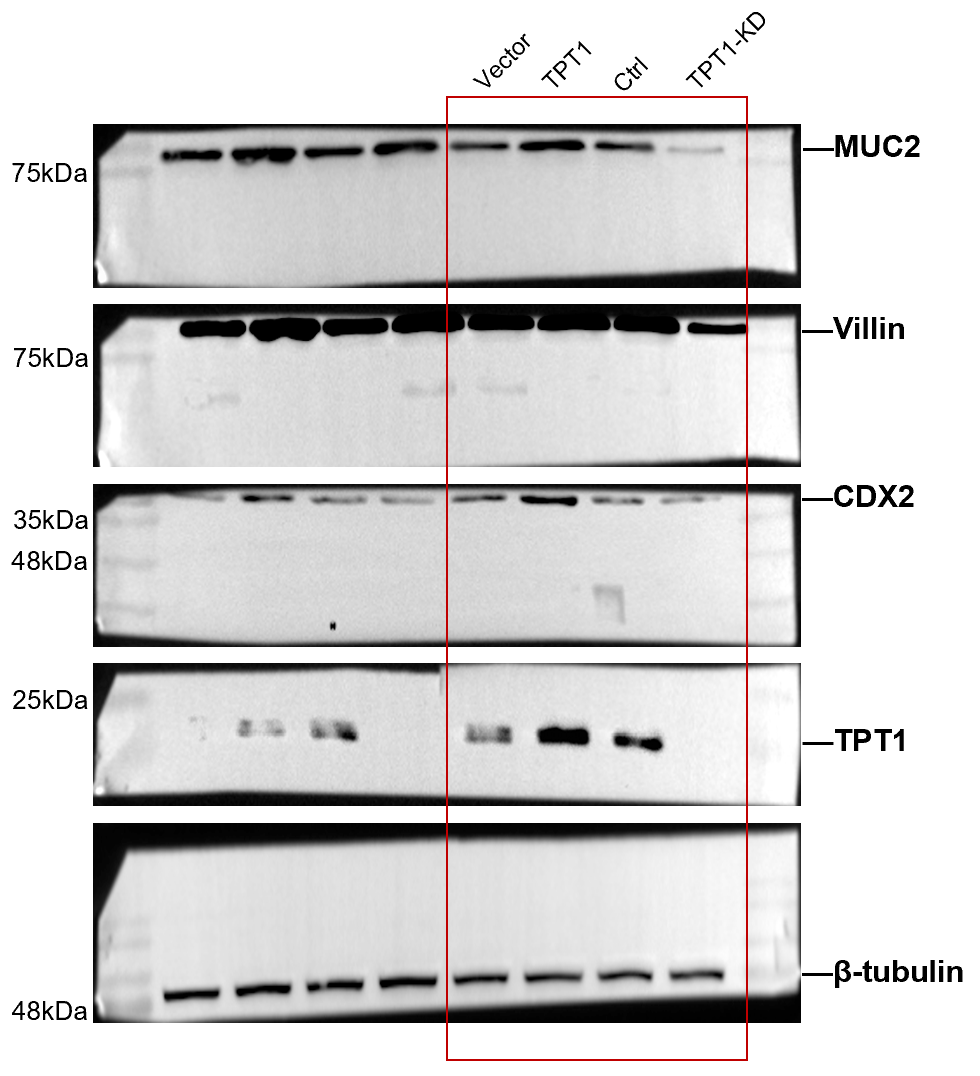
**

**Figure 4H.**

**
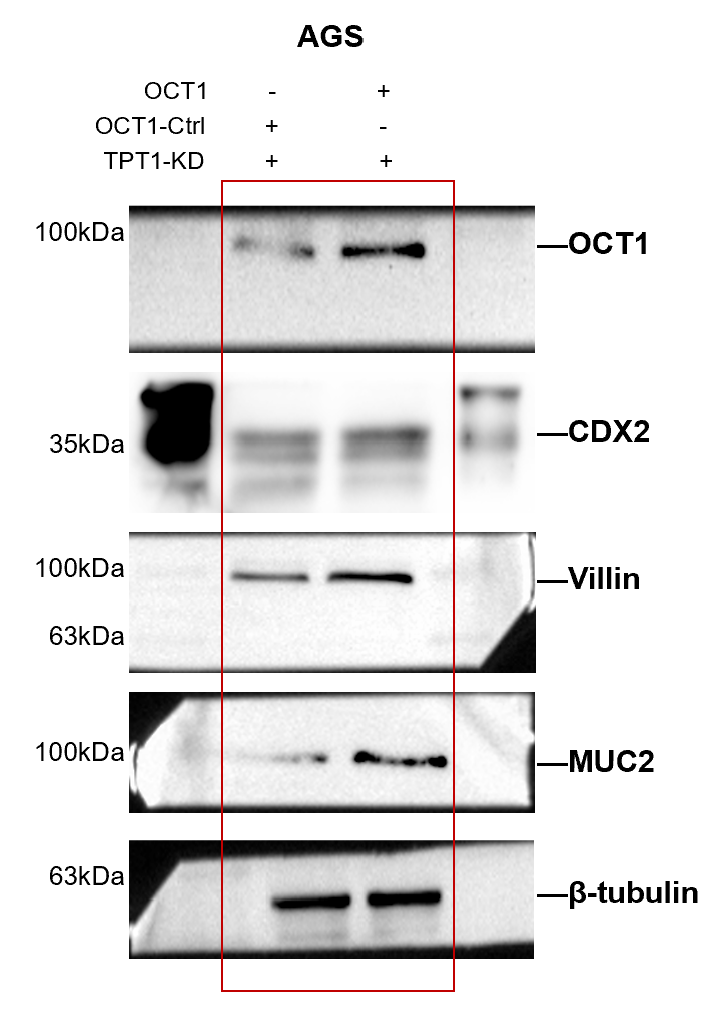
**

**Figure 5D.**

**
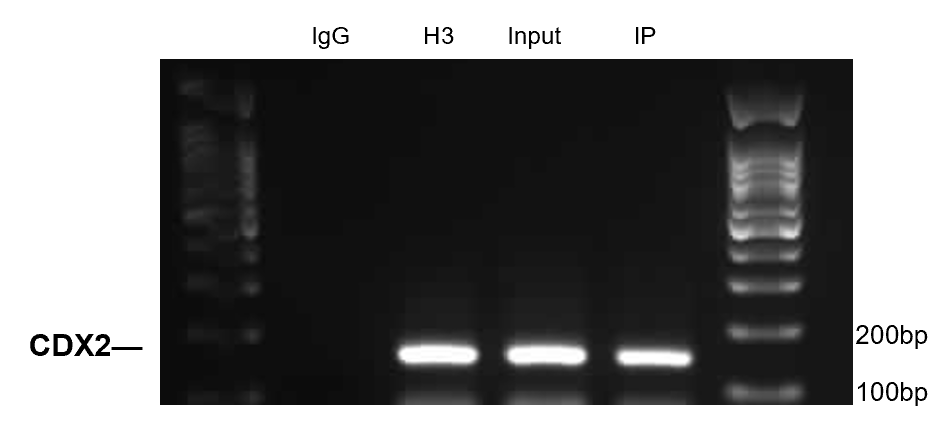
**

**Figure 6C.**

**
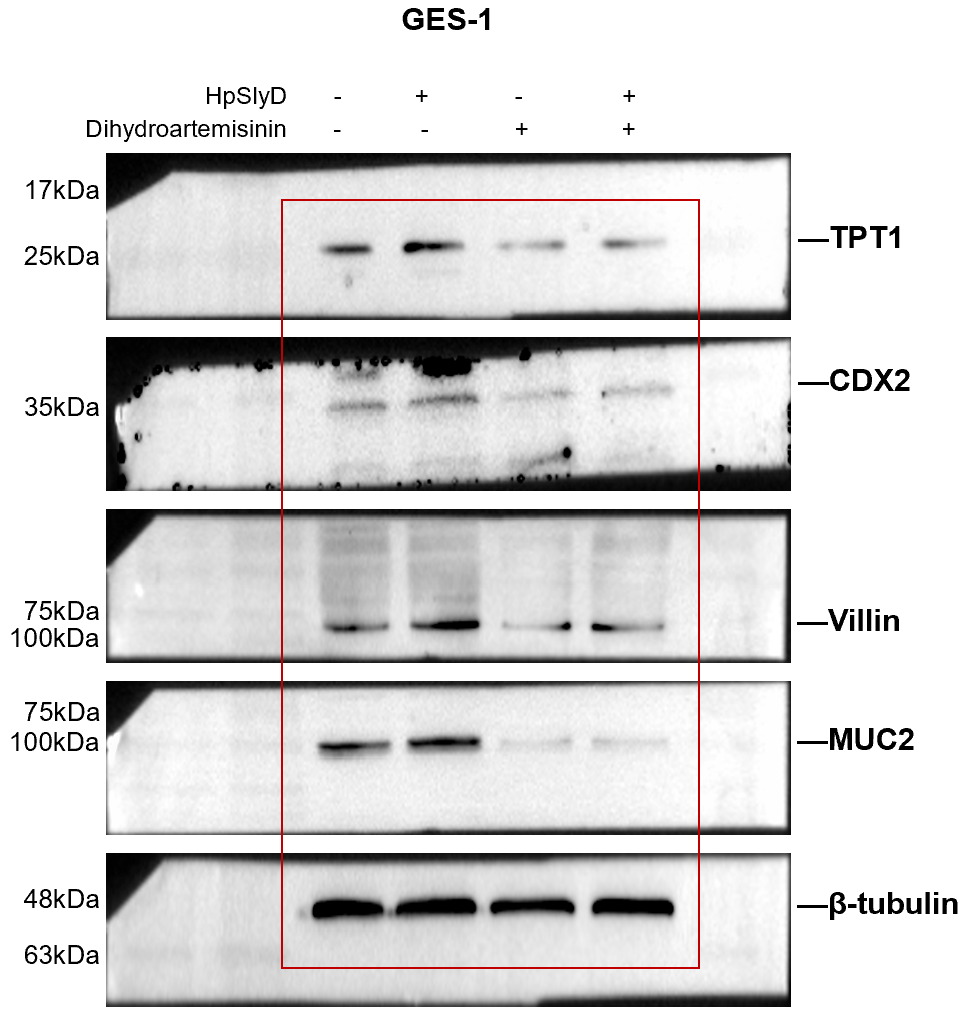
**

**Figure 6D.**

**
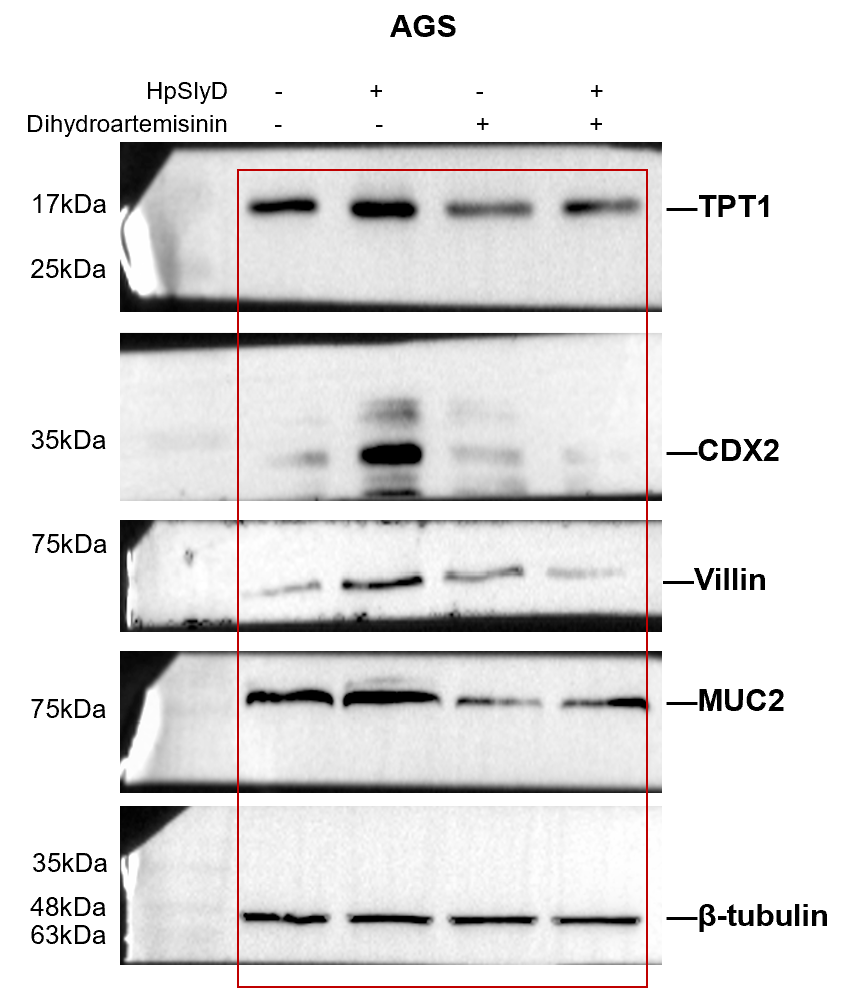
**
